# Efficient extraction of carboxylated nanocellulose from ionoSolv pulps with alkaline H_2_O_2_ assisted oxidation

**DOI:** 10.1007/s10570-024-06319-4

**Published:** 2024-12-11

**Authors:** Aida Abouelela Rafat, Pedro Verdía Barbará, Asim Ullah, Eero Kontturi, Robert V. Law, Jason P. Hallett

**Affiliations:** 1 Department of Chemical Engineering, Imperial College London, South Kensington, Exhibition Road, London, SW7 2AZ UK; 2 Department of Bioproducts and Biosystems, Aalto University, P.O Box 16300, 00076 Aalto Espoo, Finland; 3 Department of Chemistry, Molecular Sciences Research Hub, Imperial College London, 82 Wood Ln, White City CampusLondon, W12 0BZ UK

**Keywords:** Nanocellulose, Cellulose nanocrystals (CNCs), Ionic liquids, Biomass, H_2_O_2_ oxidation, Ultrasonication

## Abstract

We report a simple procedure to produce carboxylated cellulose nanocrystals (CNCs) from grassy biomass (*Miscanthus X Giganteus*) using a two-step approach consisting of biomass fractionation with a protic ionic liquid followed by oxidation of the resulting cellulose-rich pulps with H_2_O_2_. The impact of the fractionation severity on the composition, structure, size, thermal stability, crystallinity, and degree of polymerization of the CNCs was evaluated. It was found that fractionation severity had a large impact on the pulp purity and its reactivity during the oxidation stage. Nevertheless, the impact on the properties of the final CNCs was small. CNCs were recovered as suspensions of negatively charged, electrostatically stable, needle-like CNCs with a lower degree of crystallinity (58–61%) compared to the precursor pulps (65–69%). The presence of carboxyl groups on the surface of the CNCs facilitated the stability of the suspensions but also caused a slight decrease in the thermal stability of the CNCs. A milder oxidation process followed by ultrasonication allowed us to maximize the production of CNCs while better preserving the degree of crystallinity of the cellulose (63%).

**Supplementary Information:**

The online version contains supplementary material available at 10.1007/s10570-024-06319-4.

## Introduction

Cellulose is the most abundant natural biopolymer and could play a key role in the substitution of fossil fuels for the obtention of materials from renewable and sustainable sources (Li et al. 2021). Cellulose isolation from the complex hierarchical lignocellulosic biomass matrix requires the fractionation of the material using mechanical or chemical disintegration methods (Sixta 2006; Brandt et al. 2013). Recently, the isolation of cellulosic nanomaterials (CNMs) has attracted growing attention, particularly for microfibrillated cellulose (MFC), cellulose nanofibers (CNFs), nanocrystals (CNCs) or spherical nanoparticles (CNPs) (Eichhorn et al. 2010; Nechyporchuk et al. 2016). Their nanoscale particle size gives them unique properties such as large surface area, tuneable morphology (which plays a role in the surface hydrophobicity and hydrophilicity), low density, high specific strength and reactivity (Klemm et al. 2011; Foster et al. 2018). The obtention of CNMs from lignocellulosic biomass requires two separate processes: (i) a process that allows for the extraction and purification of cellulose substrates from wood or plants and, (ii) a separate process that allows for the separation and disintegration of the purified cellulose fibers into their microfibrillar and/or crystalline components (Moon et al. 2011; Brinchi et al. 2013).

Usually, the extraction and purification of cellulose from the lignocellulosic feedstocks is accomplished by means of industrial processes such as the well-established Kraft and Acid Sulphite processes or by biomass fractionation approaches such as steam explosion and dilute acid hydrolysis. The purity of the cellulose generated by these processes is typically limited, rarely exceeding 85% glucan. Further purification is usually achieved by hot or cold caustic extraction followed by a multi-step bleaching process to remove residual hemicellulose and lignin to obtain high purity cellulose (> 90% glucan) (Liu et al. 2016). Furthermore, current industrial pulping processes, based on the Kraft and Acid Sulphite technologies, are of environmental concern due to emissions of hazardous volatiles, including methyl mercaptan, hydrogen sulfide, nitrogen oxides, etc., and contaminated wastewaters containing heavy metals, nitrates, sulphates, etc. (Mandeep et al. 2019; Singh et al. 2022). Hence, alternatives are needed. Ionic liquids (ILs, salts with low melting points) have emerged as a potentially greener alternative to fractionate lignocellulose. In particular, fractionation with the ionoSolv process, based on low-cost protic ILs, allows the production of high purity cellulose-pulps in an environmentally friendly and cost-effective way (Brandt-Talbot et al. 2017; Nakasu et al. 2022). The negligible vapour pressure of most ILs, together with the possibility of recycling them without losing efficiency, allows for more environmentally friendly biomass processing, as has been assessed by cradle-to-grave life cycle analysis (Brandt-Talbot et al. 2017; Baaqel et al. 2020).

The second stage for CNMs isolation is the effective extraction of nanocellulose from the purified cellulose fibrils. The most common processes to obtain MFC or CNFs involve the use of mechanical homogenization (Shak et al. 2018), whereas CNCs/CNPs extraction is typically accomplished by acid hydrolysis or oxidation with TEMPO (2,2,6,6-tetramethylpiperidine-1-oxyl)-mediated oxidation (Rånby 1952; Pääkkönen et al. 2019; Zhang et al. 2020). However, extraction methods suffer from complex treatments and multiple purification steps, prolonged reaction times, high energy consumption or the production of large amounts of challenging waste streams (Su et al. 2022). E.g., bromide free and electromediated TEMPO oxidations required reaction times > 48 h (Brinchi et al. 2013); while CNCs extraction using acid hydrolysis requires 9 kg of H_2_SO_4_, which yields 12 kg of Na_2_SO_4_ after acid neutralization, per kg of CNCs produced (Chen et al. 2016; Gupta and Shukla 2020). These factors hinder the progress towards efficient process scale-up and commercial use of CNCs.

Recent methods developed to overcome these limitations include hydrolysis with dicarboxylic acids (Chen et al. 2016; Wang et al. 2017), citric acid (Ji et al. 2019) and ILs (Haron et al. 2022; Paredes et al. 2022); or the use of oxidizing agents as ammonium persulfate (APS) (Leung et al. 2011; Cheng et al. 2014) and H_2_O_2_ (Koshani et al. 2018). Different protic ILs have been used to produce CNCs from a variety of cellulosic feedstocks, including micro crystalline cellulose (MCC), commercial cellulose, *Miscanthus* or corn husk, using a variety of conditions with times and temperatures ranging from 15 min to 24 h and 60 to 120 °C (Paredes et al. 2022). The use of a protic IL with anion clusters containing sulfuric acid ([Hmim][(HSO_4_)(H_2_SO_4_)]_x=0,1,2_), allowed for yields of 70% under milder conditions (3 h at 40 °C) (Paredes et al. 2022). Mixtures of aprotic ILs with organic solvents, needed to swell the MFC and decrease the viscosity of the IL, have been also reported recently (Haron et al. 2022). Alkaline oxidation with H_2_O_2_ -an oxidizing agent commonly used in the pulp and paper industry as an environmentally friendly chlorine-free bleaching alternative- has been successful in the extraction of CNCs at lab-scale. However, the processing conditions (solid to liquid ratio of 1:20 g g^−1^ and particle sizes ≤ 60-mesh) are economically unfeasible when considering large scale CNC production (Li et al. 2016). The use of copper catalysts for CNC isolation with H_2_O_2_ has been also reported, but this requires extremely long reaction times (72 h) and large reactor volumes, which counterbalances the benefits of using H_2_O_2_ (Koshani et al. 2018).

Here, we explore a simple procedure to produce carboxylated CNCs from unbleached cellulose pulps, obtained by ionoSolv fractionation process followed by a 1 h alkaline H_2_O_2_ oxidation. Since cellulosic substrates are known to have certain degree of resistance to oxidation due to high crystallinity and low accessibility to reagents, an alkali NaOH solution was used in conjunction with H_2_O_2_ oxidation to improve the cellulose pulp accessibility via a fibre-swelling phenomenon (Pönni et al. 2014; Budtova and Navard 2016). The two-step process involved the use of the low-cost *N*,*N* -dimethyl-*N*-butylammonium hydrogen sulfate, [DMBA][HSO_4_], to fractionate *Miscanthus* × *giganteus* (particle size of 1–3 mm) to produce cellulose-rich pulps followed by alkaline H_2_O_2_ oxidation of the obtained pulps at solid loading of 1: 10 g g^−1^ to produce (i) a highly pure, low degree of polymerization (DP) cellulose residue that can be further tailored and processed to produce CNFs or CNCs and (ii) a functionalized carboxylated CNC suspension. We investigated the impact of controlling ionoSolv fractionation severity on the cellulose (pulp and oxidation residue) composition, crystallinity and degree of polymerization as well as on the properties of the carboxylated CNCs in terms of structure, size and stability. We showed that fractionation severity during ionoSolv biomass fractionation had a significant impact on the cellulose-rich pulp as well as on the alkaline H_2_O_2_ cellulose residue in terms of chemical structure and degree of polymerization. However, the fractionation conditions had only a minor impact on the properties of the carboxylated CNCs produced. Furthermore, to achieve complete conversion of the bleached pulps into CNC, an ultrasonication stage was applied to the solid fraction that did remain as a solid bleached cellulose residue after the oxidation with H_2_O_2_ (Metzger et al. 2021).

This is the first study to demonstrate the successful production of functionalized negatively charged carboxylic-CNCs from ionoSolv cellulose pulps.

### Hypothesis

Functionalized CNCs and near levelling-off degree of polymerization (LODP) cellulose can be produced from lignocellulosic biomass using acidic protic ionic liquid fractionation followed by alkaline oxidation.

## Materials and methods

### Materials

*Miscanthus x giganteus* was acquired from Silwood Park campus, Imperial College London. Starting materials for ionic liquid synthesis and alkaline H_2_O_2_ oxidation method were purchased from Sigma Aldrich and, unless stated otherwise, used as received. Market and commercial pulps were obtained from a commercial pulp supplier.

### Ionic liquid synthesis

*N,N*-dimethyl-*N*-butylamine (101.19 g, 1 mol) was added into a 500 mL round-bottom flask and cooled in an ice bath. 200 mL of 5 M H_2_SO_4_ (1 mol) were added dropwise using a funnel while stirring. The reaction proceeded for at least 5 h with continuous stirring. Excess water was removed by heating at 40 °C under reduced pressure. The IL was recovered as a clear, viscous liquid. The water content of the IL was adjusted to 20 wt% as measured using Volumetric Karl Fisher Titrator (V20 Mettler-Toledo). ^1^H NMR δH (400 MHz, DMSO-d_6_)/ppm: 9.24 (s, 1H, N–*H*), 3.02 (dt, J = 12.9, 5.0 Hz, 2H, N–C*H*_*2*_), 2.76 (d, J = 4.3 Hz, 6H, N–(C*H*_*3*_)_2_), 1.64–1.51 (m, 2H, N–CH_2_–C*H*_*2*_), 1.30 (h, J = 7.4 Hz, 2H, N–(CH_2_)_2_–C*H*_*2*_), 0.90 (t, J = 7.4 Hz, 3H, N–(CH_2_)_3_–C*H*_*3*_). ^13^C NMR δC (101 MHz, DMSO-d_6_)/ppm: 55.92 (N–*C*H_2_), 42.46 (N–*C*H_3_), 25.82 (N–CH_2_–*C*H_2_), 19.30 (N–(CH_2_)_2_–*C*H_2_), 13.71 (N–(CH_2_)_3_–*C*H_3_).

### Biomass fractionation

Pretreatment was conducted according to the standard operating procedure from our laboratory (Gschwend et al. 2016). Determination of oven dried weight of biomass and cellulose pulps (air-dried and wet conditions) was conducted according to the NREL (National Renewable Energy Laboratory) protocol “Determination of Total Solids in Biomass and Total Dissolved Solids in Liquid Process Samples” (Sluiter A et al. 2008). In short, the IL solution was mixed with 2 g of biomass (on an oven-dried weight basis) in a pressure tube, corresponding to a biomass to solvent ratio of 1:5 g g^−1^, with the solvent being a mixture of 80 wt% of N,N-dimethylbutylammonium hydrogen sulfate and 20 wt% water ([DMBA][HSO_4_]_80%_). The pretreatment was conducted at oven temperature of 120 °C for 6 h and 170 °C for 45 min, respectively. In this document, the cellulosic pulps recovered after the pretreatments at 120 °C and 170 °C will be referred as M120 and M170, respectively. A detailed description of the procedure can be found in the Supplementary Information (SI).

### Alkaline H_2_O_2_ oxidative preparation of CNCs

Air dried *Miscanthus* pulp (5 g) was added to 250 mL in a three-neck round-bottom flask and mixed with 10 wt% NaOH solution (25 mL) for 15 min at 80 °C using a heating plate, as monitored by a thermometer inside the flask. After 15 min of stirring, 30 wt% H_2_O_2_ solution (25 mL) was added carefully dropwise using a volumetric funnel and the reaction proceeded for 45 min, counting from the start of the addition. The drop-wise addition of H_2_O_2_ needs to be continuously monitored during the reaction course. The round-bottom flask was covered with aluminium foil and the reaction mixture was constantly stirred at 100 rpm. After the process was completed, the final volume of the suspension was adjusted to 200 mL with cold deionized water to terminate the reaction. The resulting product mixture contained the cellulose residue solids -which will be referred to as BM120 and BM170 from now on, with the numbers corresponding to the temperature of the initial pretreatment employed for each sample- and the CNC particles in suspension -which will be referred to as NBM120 and NBM170 following the same criteria as for the ionoSolv and the bleached pulps- and was centrifuged at 4000 rpm for 30 min. The first supernatant fluid was discarded to remove NaOH/H_2_O_2_ reaction mixture. The cellulose residue solids were then repeatedly washed with DI water and centrifuged at 4000 rpm for 30 min until the pH reached 8 (samples named as BM120 and BM170). The successive decanted washings contained CNC particle suspensions. These were collected and dialyzed against deionized water. A small fraction of the dialyzed CNC suspension was freeze-dried for analysis. The remaining CNC suspension was stored in a refrigerator at 4 °C.

### Bleaching of pulps by mild akaline H_2_O_2_ oxidation

Air dried pre-treated *Miscanthus* × *giganteus* pulp was weighed into a medium-sized Ziplock bag contained inside two more medium-sized Ziplock bags. A solution containing 0.2 wt% relative to the pulp of diethylenetriaminepentaacetic acid (DTPA) as a chelating agent and 5 wt% relative to the pulp of NaOH in deionized (DI) water was added. The total amount of DI water to be employed was calculated so the final pulp consistency after the addition of the H_2_O_2_ solution equalled 10 wt%. The mixture was heated at 80 °C in a water bath and a H_2_O_2_ solution was added so the final H_2_O_2_ amount equalled 4 wt% relative to the pulp. The exact concentration of the H_2_O_2_ solutions was determined before each experiment using the appropriate protocol (2008). The mixture was mixed by hand every 10 to 15 min for the first hour, every 20 to 30 min for the second hour, and every 30 min after the second hour. After 3 h the reaction was stopped by placing the mixing bag into an ice bath and the liquor was filtered off. 2 samples of the filtrate were taken, one to determine the final pH and a second one to determine the amount of H_2_O_2_ consumed. The bleached pulps were collected, washed with DI water, acidified to pH in the range between 4 and 5 with a sodium bisulphite solution, filtered and washed with DI water again. Finally, the pulps were weighed, and the moisture level was measured.

### Chemical compositional analysis

Compositional analysis was carried out according to the published procedure ‘Determination of Structural Carbohydrates and Lignin in Biomass’ by NREL (Sluiter et al. 2012). The detailed procedure can be found in the SI. Compositional analysis determines the following structural components: (i) carbohydrates content, namely glucose, xylose, arabinose and mannose, (ii) acid insoluble lignin, (iii) acid soluble lignin, (iv) acid insoluble ash. A Prominence HPLC system (Shimadzu) with refractive index (RI) detector was used to quantify the sugars, employing an Aminex HPX-87P column operated at 85 °C with purified water at a flow. A detailed procedure description can be found in the SI.

The glucose content determined using this protocol does not distinguish between glucose derived from cellulose or the glucose derived from hemicellulose. Therefore, the glucan content in the pulp and untreated biomass is taken as the total glucose derived from both cellulose and hemicellulose, whereas hemicellulose content in the residue and untreated biomass is taken as the sum of all carbohydrate sugars (xylose, galactose, arabinose, mannose) except glucose.

Samples of dissolving and commercial pulps and bleached *Miscanthus* were independently analysed by Celignis Analytical using an infrared (IR) methodology.

### Gel permeation chromatography (GPC) analysis

The ionoSolv and bleached pulps were analysed for their molar weight distribution at the Department of Forest Products Technology at Aalto University, Finland. Prior to the analysis, the samples were ground to particles smaller than 60 mesh size and delignified with sodium chlorite aqueous solution. The delignified samples were pre-activated by a water/acetone/*N*,*N*-dimethylacetamide (DMAc) solvent exchange sequence. The samples were then dissolved at 90 g/L in a lithium chloride (LiCl)/DMAc mixture at room temperature. The solutions were diluted to 9 g/L LiCl/DMAc and filtered through a 0.2 mm syringe filter and analyzed with a Dionex Ultimate 3000 system with RI detection (Shodex RI-101). Average degree of polymerization (DP_average_) was calculated by dividing the average molecular weight (M_w_) by the molecular weight of the anhydrous glucose unit (162 g/mol).

### X-ray diffraction (XRD) and crystallinity analysis

XRD patterns were collected with Xpert pro PANalytical (Malvern Panalytical, Spectris UK) X-ray operated at 40 kV and 40 mA with a CuK_α_ radiation source at room temperature. The scattering angle range was 5 − 45° 2θ at a scanning speed of 70 s per step and a 2θ step interval of 0.02°. The crystallinity index (CrI) was calculated following Segal method (Segal et al. 1959) using the Equation:

$$CrI=\left({I}_{200}-{\text{I}}_{am}\right)/{I}_{200 }\text{ x}100$$

This method has been extended according to the parameters published in literature, so it can be applied to calculate the CrI for Cellulose II (Azubuike et al. 2012; French and Santiago Cintrón 2013; Nam et al. 2016). Here, I_200_ is the intensity of the peak at (I200, 2θ = 22.7° for cellulose I and I020, 2θ = 21.7° for cellulose II)°, assigned to the crystalline portion of cellulose, and I_am_ is the intensity at 2θ = 18° for cellulose I and 2θ = 16° for cellulose II which is assigned as the crystallite defect portion of cellulose. No background correction was made.

### Fourier transform infrared (FT-IR) spectroscopy

Samples were characterized with an FT-IR spectrometer using Perkin-Elmer with a diamond attenuated total reflectance (ATR) single reflection crystal. Initial background spectra were taken and subtracted from the sample measurement. Solid samples were placed directly on the ATR crystal, and maximum pressure was applied by lowering the tip of the pressure clamp using a rachet-type clutch mechanism. All the spectra of measured samples were averaged from 32 scans from 550 to 4000 cm^−1^ with a resolution of 2 cm^−1^.

### Zeta potential (ZP) analysis

Zeta potential values were determined using the Brookhaven ZetaPals (BrookHaven Instruments, Nova instruments, USA). The measurements were conducted in triplicate for each sample, with 10 runs per sample and 15 cycles per run and the average value is reported. All measurements were conducted in DI water at a pH of 6.5.

### Morphological studies of CNCs

Scanning electron microscopy (SEM) images were taken by Dr. Ecaterina Ware, procedure Department of Materials, Imperial College London, using a SEM JOEL 6010A microscope. Samples were coated with a thin layer of gold and images were captured up with 10 kV accelerated voltage. Atomic Force Microscopy (AFM) was conducted using AFM metal disc glued by freshly cleaved mica and coated with (3-Aminopropyl)triethoxysilane (APTES) using the vapor-phase method 0.01 wt%. TEMPO-oxidized cellulose nanofibril (TOCNF) was spin-coated on the surface-modified metal.

### Ultraviolet–visible (UV–vis) spectroscopy

The optical transmittance of CNCs suspensions (0.1 wt%) were measured using Shimadzu UV-1800 UV spectrophotometer. A background spectrum was first acquired from the empty sample holder. The spectra were acquired from 200 to 900 nm with a data interval of 1 nm.

### Thermogravimetric analysis (TGA)

TGA on freeze-dried CNC samples was performed using TA Instruments Q500 TG analyzer. Samples (about 5 mg) were heated in a pure nitrogen atmosphere (flow rate 60 mL/min) from 25 to 600 °C at a rate of 10 °C/min.

### Solid state ^13^C nuclear magnetic resonance (NMR)

Solid-state ^13^C NMR spectra were recorded on a Bruker Avance 200 MHz spectrometer operating at 50.1 MHz (4.7 T) at room temperature using proton dipolar decoupling, magic angle spinning (MAS) and ramp cross-polarisation. The ^1^H radio rf strength was set to give a 90° pulse duration ca. 3.5 µs. The recycle delay for the cross-polarisation was 5.0 s. The contact time was 1.0 ms. The ramp was set between 70 and 100% throughout the contact time. The samples were carefully ground and place in a 4 mm zirconia rotor and spun at 5.0–7.0 kHz. The chemical shift values were measured with respect to tetramethylsilane via tetrakistrimethylsilylsilane reference with the methyl signal set at 3.52 ppm.

### Production of CNCs by ultrasonication

The bleached pulps were placed in a glass pressure tube, diluted with DI water to the top with the ultrasonication probe placed inside the solution, and then ultrasonicated using a frequency of 20 kHz at 120 W and 60% power for different intervals (15 min, 30 min and 1 h) in an ultrasonic crusher to get an aqueous homogenized dispersion.

## Results and discussions

### Cellulose characterization

#### Chemical compositional analysis (CA)

**Fig. 1:**
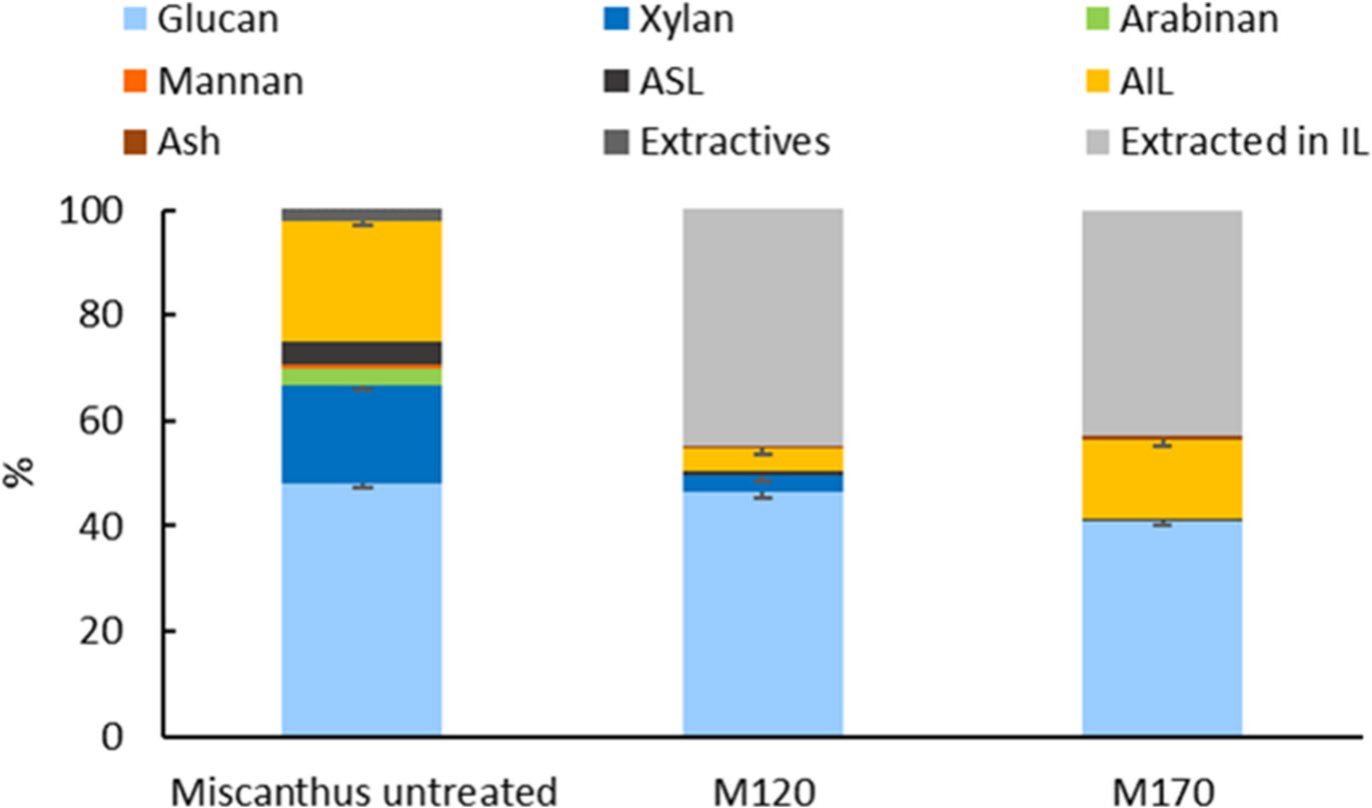
Composition of untreated *Miscanthus* and *Miscanthus* cellulose pulps at 120 °C for 6 h (M120) and for 170 °C for 45 min (M170). The cellulose pulp composition values were normalized using the gravimetric pulp yield. ASL stands for Acid Soluble Lignin while AIL stands for Acid Insoluble Lignin, according to the published procedure ‘Determination of Structural Carbohydrates and Lignin in Biomass’ by NREL (Sluiter et al. 2012). Fractionation experiments were conducted using [DMBA][HSO_4_]_80%_ at 20 wt% solids loading

The effect of *Miscanthus* fractionation by [DMBA][HSO_4_]:H_2_O = 80:20 wt% mixture ([DMBA][HSO_4_]_80%_) was quantified by analysing the structural compositions of the feedstock and cellulose pulps. Fractionation experiments were conducted using [DMBA][HSO_4_]_80%_ at two conditions: 170 °C for 45 min (high severity) and 120 °C for 6 h (low severity). Cellulose pulp samples were labelled according to the fractionation conditions as **M120** and **M170**, such that *Miscanthus* pulp from ionoSolv treatment at 120 °C for 6 h is indicated as **M120**. The letter **B** is used to indicate the corresponding post-treated treatment alkaline H_2_O_2_ (bleached) cellulose residues **BM120** and **BM170**. Commercial grade dissolving and market pulps were also analysed for their structural compositions and compared to the quality of the cellulose pulps. Dissolving pulp is a high-grade cellulose pulp with cellulose content > 90%, typically used for textile and regenerated cellulose applications; whereas market pulp is a lower-grade cellulose pulp used for paper applications (Liu et al. 2016). Table 1 presents the numerical values of the glucan, hemicellulose, Klason lignin and ash contents for the untreated *Miscanthus*, ionoSolv protic IL treated cellulose pulps and the alkaline H_2_O_2_ cellulose residues. Figure 1 shows the structural compositions of the untreated *Miscanthus* as well as the cellulose pulps obtained from the fractionation using [DMBA][HSO_4_]_80%_.

**Table 1:** Structural composition of untreated *Miscanthus*, ionoSolv cellulose pulps, ionoSolv + alkaline H_2_O_2_ oxidized cellulose residues, dissolving pulp and market pulp. IonoSolv fractionation experiments were conducted at 120 °C for 6 h (M120) or 170 °C for 45 min (M170) using [DMBA][HSO_4_]_80%_ and 20 wt% solids loading. Alkaline H_2_O_2_ oxidation were conducted using 10 wt% NaOH and 30 wt% H_2_O_2_ at 80 °C and 10 wt% solids loading. Yields of M170 and M120 are given relative to the amount of *Miscanthus* used in the pretreatment. Yields of BM170 and BM120 are relative to the amount of M170 and M120, respectively, used in the bleaching experiments

Sample	Yield	Glucan	Hemicellulose	Lignin	Ash	Extractives
*Miscanthus* untreated	–	48.2	21.9	27.3	1.1	1.5
M170	46.3	66.6	–	31.6	1.8	–
M120	47.2	84.4	5.1	9.2	1.3	–
BM170	88.7	93.2	-	5.1	1.7	–
BM120	74.9	93.3	1.3	4.8	0.6	–
Dissolving pulp	–	92.8	0.4	5.3	1.5	–
Market pulp	–	85.0	7.2	6.7	1.1	–

It was interesting to note that the lower severity conditions resulted in higher delignification, with 81% delignification achieved for M120 (log R_0_^*^ = 1.50) compared to only 44.5% for M170 (log R_0_^*^ = 2.07) (Abouelela et al. 2023). The higher lignin content of 31.6% in M170 compared to the 9.2% lignin content in M120 is likely related to the rapid delignification and subsequent pseudo lignin formation kinetics in [DMBA][HSO_4_]_80%_ at 170 °C compared to 120 °C, as shown in previous studies (Gschwend et al. 2018). Pseudo lignin formation can be attributed to recondensation and repolymerization reactions of extracted lignin fragments and polysaccharide degradation products (Gschwend et al. 2018; Shinde et al. 2018). The fast and higher likelihood of pseudo lignin deposition on cellulose pulp surface at high severity fractionation conditions increased the overall Klason lignin content in cellulose pulp where it exceeds the lignin content in the untreated biomass (Table 1) (Shinde et al. 2018). Figure SI-1 shows the impact of the fractionation conditions on the cellulose pulp brightness, with M120 showing lighter colour than M170.

Producing hemicellulose-lean cellulose pulps is of key significance in cellulose pulp processing, particularly to produce dissolving pulp, regenerated cellulose products and cellulose derivatives, since hemicellulose interferes in the regeneration process of cellulose due to its chemical similarities (Froschauer et al. 2013). Currently, wood-derived dissolving pulps are produced by acid sulphite or pre-hydrolysis kraft processes. Effective removal of hemicellulose in both processes is insufficient and, therefore, subsequent hot and/or cold caustic soda extractions steps are integrated to achieve the required extraction (Liu et al. 2016). On the other hand, effective removal of hemicellulose is one of the key features of ionoSolv process, due to the acidic nature of the protic IL [DMBA][HSO_4_]. It promotes the depolymerization and hydrolysis of hemicellulose into monomeric sugars (e.g., xylose, mannose), oligomers and their dehydration products (furfural, 5-(hydroxymethyl)furfural (HMF)) (Brandt-Talbot et al. 2017). Removal of hemicellulose was more effective at higher severity conditions with quantitative removal achieved for M170, while a lower hemicellulose removal of 87% was obtained for M120. However, the effective hemicellulose removal at high severity pretreatment was compromised by the higher degradation of glucan in M170, a loss of 15.1% relative to the initial content. Lower severity fractionation preserved the glucan more intact with loses of only 3.8% for M120.

**Fig. 2:**
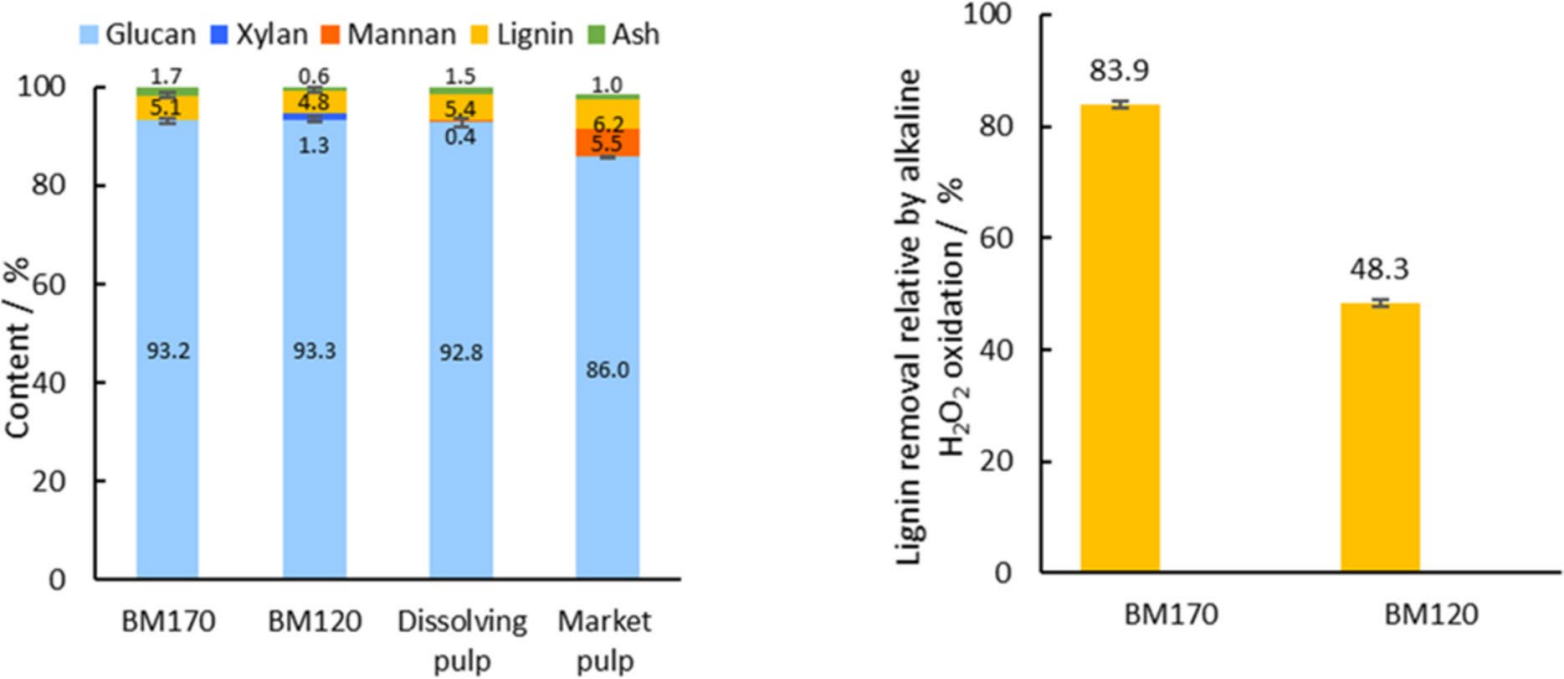
**a** Structural composition of commercial grade dissolving pulp, market pulp and the alkaline H_2_O_2_ cellulose residues BM170 and BM120, **b** Lignin removal by alkaline H_2_O_2_ oxidation relative to lignin content in ionoSolv cellulose pulps M170 and M120. Alkaline H_2_O_2_ oxidation was conducted at 80 °C using 10 wt% NaOH and 30 wt% H_2_O_2_ using 10 wt% solids loading

Figure 2a presents the structural carbohydrate, lignin, and ash composition of the cellulose residues BM120 and BM170 after the alkaline H_2_O_2_ oxidation, which resulted in a significant improvement in the purity of the cellulosic pulp by further removing hemicellulose and residual lignin. The structural composition of the alkaline H_2_O_2_ cellulose residues BM120 and BM170 showed higher purity of glucan (> 90%) compared to commercial market pulp quality and a comparable glucan and lignin composition to dissolving pulp. This highlights that effectiveness of the alkaline H_2_O_2_ in removing residual lignin to very low levels to an extent similar to that of commercial bleaching processes. It should be noted that the commercial pulps employed here for comparison purposes, namely dissolving pulp and market pulp, were bleached using an Elemental Chlorine Free (EFC) process that employs an oxygen delignification step followed by treatments with both chlorine dioxide and H_2_O_2_ as bleaching agents to reduce lignin content. H_2_O_2_ treatment for cellulose bleaching is typically employed as the last stage of a sequence of bleaching stages that involves a first stage based on oxygen delignification, followed by an alkaline treatment, before the final H_2_O_2_ bleaching stage. This sequence improves the delignification (lignin content < 5%) (Major et al. 2005; Sousa et al. 2023). BM120 had the lowest amount of residual lignin of 4.8% with a cellulose content of 93% and hemicellulose content of 1.3%. The glucan composition of BM170 was very similar to BM120 with slightly higher lignin content of 5.1% and no hemicellulose content.

It should be noted that independent analysis of the composition of the Dissolving Pulp, Market Pulp and BM170, carried out by Celignis Analytics using a near-field infrared spectroscopy (NFIR) methodology showed that the lignin contents are in all cases lower than what was detected with CA. For Dissolving Pulp a lignin content of 1.75% was detected according the NFIR method (0.92% Klason lignin, 0.83% acid soluble lignin), vs 5.3% detected by CA; for Market Pulp the detected lignin content with NFIR was 4.08% (3.20% Klason lignin, 0.88% acid soluble lignin) versus 6.7% detected by CA; and for the bleached *Miscanthus* Pulp BM170 1.65% lignin content was detected by the NFIR method (0.99% Klason lignin, 0.66% acid soluble lignin) versus 5.1% detected by CA. This is possibly because the compositional analysis procedure, as developed by the NREL, is a standardized method based on the use of sulfuric acid and optimized for raw biomass samples with high lignin content. When employed for ultra-low lignin content pulps, the exposure to the sulfuric acid can cause degradation of a small fraction of the cellulose sugars into humins and other degradation products that show as lignin content in the analysis.

It is noticeable that lignin removal during the alkaline H_2_O_2_ oxidation from M120 cellulose pulp was significantly lower (48%) than from M170 (84%, Fig. 2b) even though M170 had a higher initial Klason lignin content (31.6%) than M120 (9.2%). The higher Klason lignin content of M170 is the result of reprecipitation of lignin that had been firstly removed from the biomass, then recondensed in solution and finally reprecipitated onto the pulp surface and of condensation and polymerization reactions of the decomposition intermediates of plant polysaccharides (Gschwend et al. 2018; Shinde et al. 2018). Our observations suggest that this pseudo lignin is easier to remove during the alkaline H_2_O_2_ oxidation as it is deposited onto the pulp surface with little, if any, involvement of lignin carbohydrate complexes (LCCs) (Shinde et al. 2018).

**Fig. 3:**
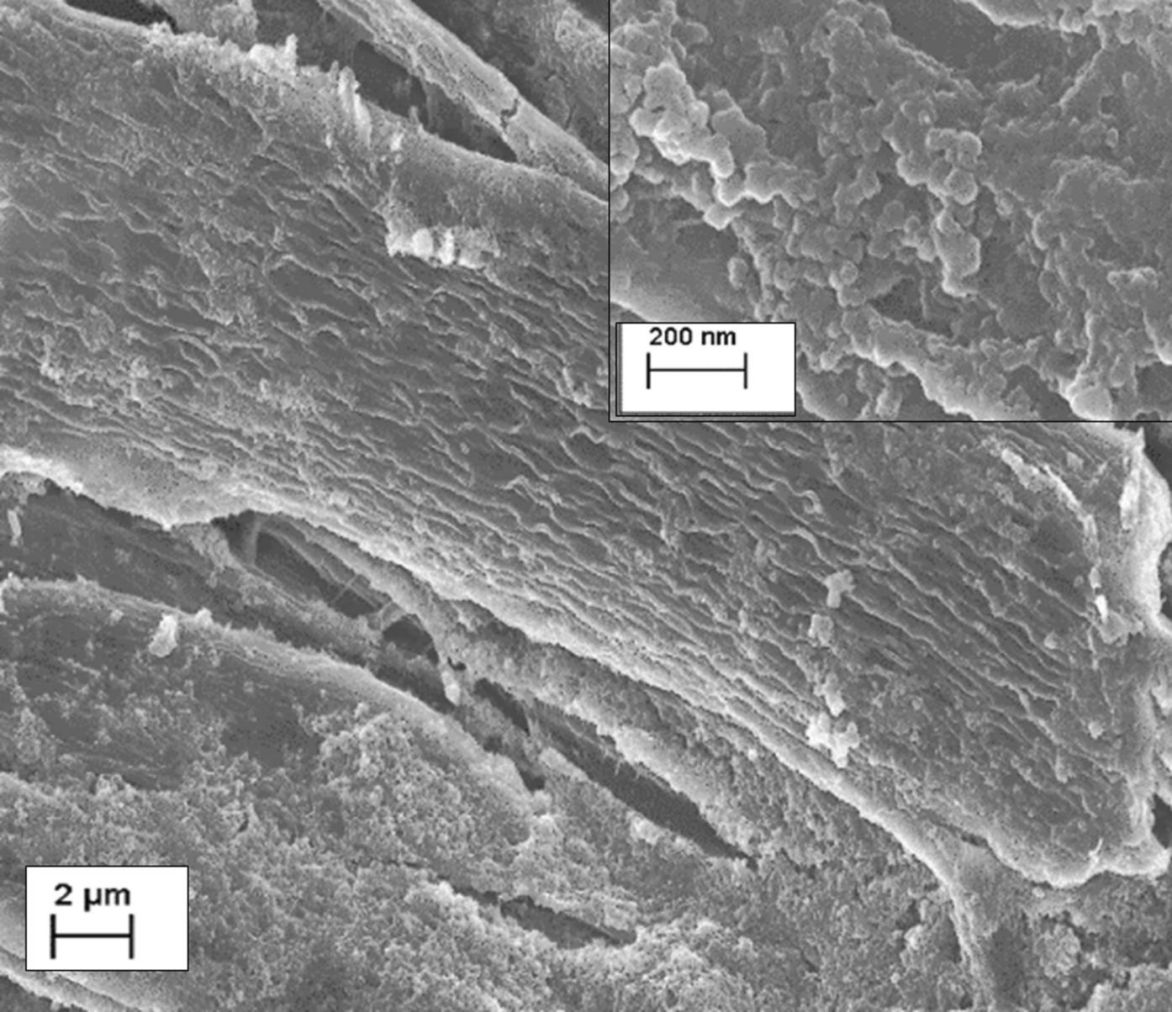
SEM image of the M170 ionoSolv treated cellulose pulp showing the pseudo lignin droplets deposited onto the cellulose fibres

Figure 3 presents the SEM image of the M170 cellulose pulp. Pseudo lignin deposition can be seen as spherical droplets on the surface of the fibres. These are typically reported to describe pseudo lignin formed during high severity dilute acid pretreatment as a result of lignin migration from the cell wall to the bulk liquid followed by its re-deposition on the cellulose surface (Shinde et al. 2018).

#### Molecular weight distribution of cellulose pulps

**Fig. 4:**
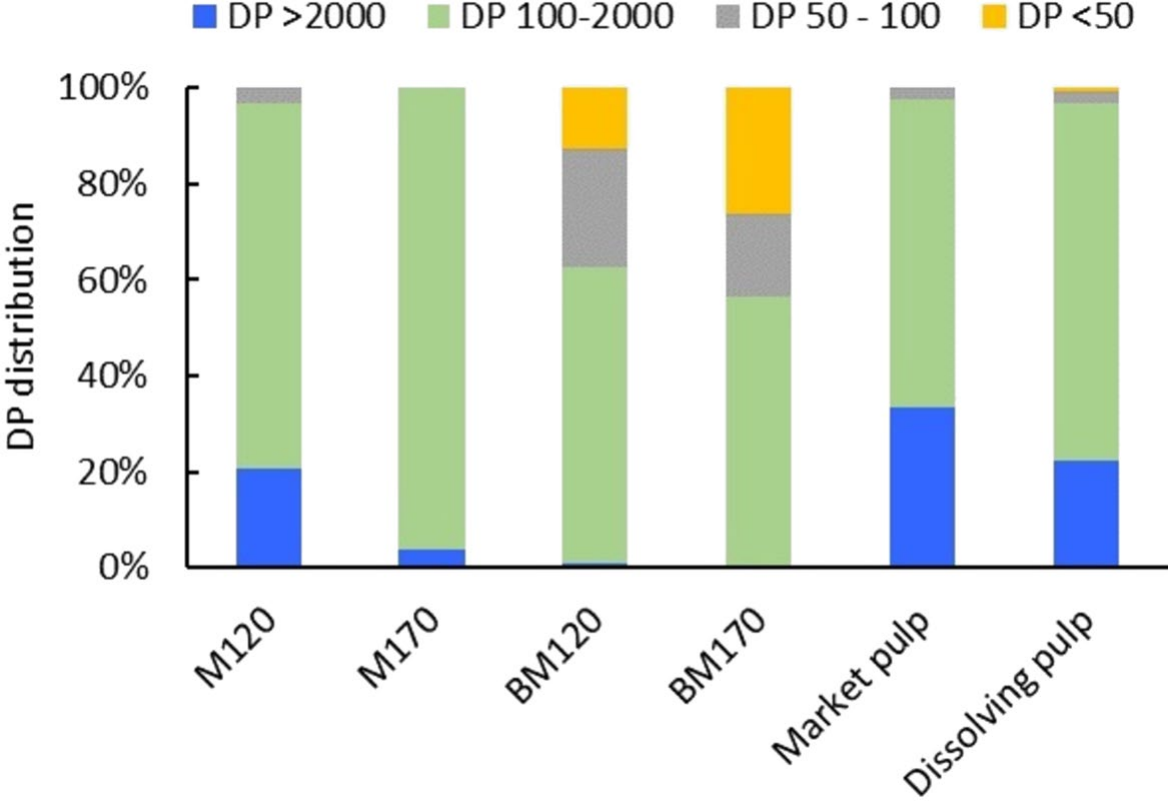
DP distribution of ionoSolv treated pulps, alkaline H_2_O_2_ cellulose residues, dissolving pulp and market pulp. Fractionation experiments were conducted using [DMBA][HSO_4_]_80%_ at 20 wt% solid loading. Alkaline H_2_O_2_ oxidation was conducted at 80 °C using 10 wt% NaOH and 30 wt% H_2_O_2_ using 10 wt% solids loading

**Fig. 5:**
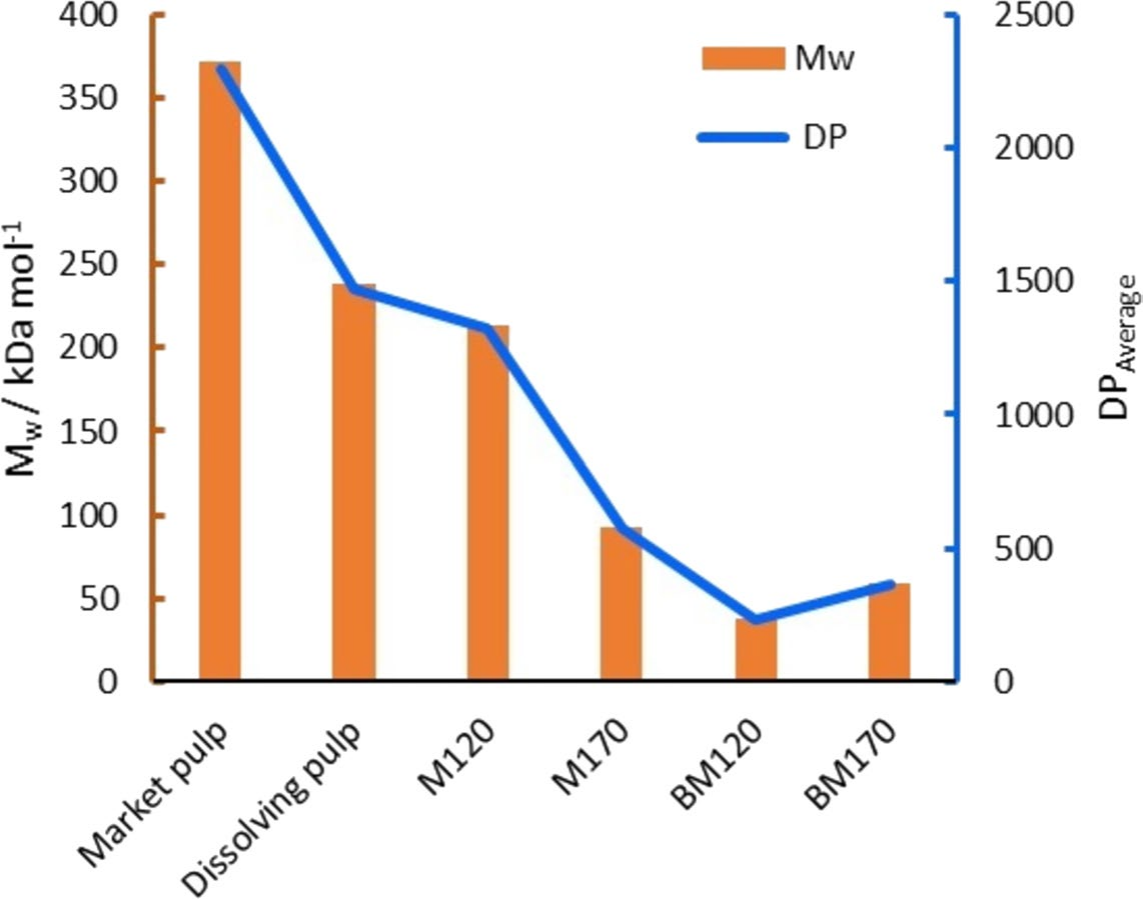
Molecular weight and average degree of polymerization of ionoSolv treated pulps and alkaline H_2_O_2_ cellulose residues. Fractionation experiments were conducted using [DMBA][HSO_4_]_80%_ at 20 wt% solids loading. Alkaline H_2_O_2_ oxidation was conducted at 80 °C using 10 wt% NaOH and 30 wt% H_2_O_2_ using 10 wt% solids loading

Figure 4 presents the DP distribution of cellulose in ionoSolv pulps and in the cellulose residues from the alkaline H_2_O_2_ oxidation using gel permeation chromatography (GPC). The M_w_ and the DP_average_ (DP_average_ = M_w_/162) are also presented in Fig. 5. Conducting the GPC analysis on the initial feedstock was not possible due to the high amount of initial lignin present that prevented the sample from dissolving as part of the sample preparation procedure. During the [DMBA][HSO_4_]_80%_ ionoSolv fractionation process, the biomass feedstock is subject to highly acidic conditions (Gregorio et al. 2016). It is well known that when cellulose is subjected to acidic conditions, the glucosidic linkages in the cellulose fibres are broken and the DP decreases (Gregorio et al. 2016; Tu et al. 2020). For ionoSolv treated pulps, *Miscanthus* treated cellulose pulps produced at high severity condition (M170) had a substantially lower M_w_ of 93 kDa mol^−1^ (DP_average_ = 574) compared to M120 with M_w_ of 214 kDa mol^−1^ (DP_average_ = 1318). The DP distribution showed that 20% of the cellulose chains in M120 had DP > 2000 compared to only 3.8% for M170. This indicates that low severity conditions retain the high DP cellulose chains more efficiently with only minimal degradation and hydrolysis of the crystallite defects in the cellulose in the IL media as demonstrated earlier. Conversely, the high severity conditions promoted faster and more severe depolymerization and hydrolysis of both the crystallite defects of the cellulose as well as the crystalline regions, resulting in lower M_w_ and DP_ave_.

The DP distribution of the M120 ionoSolv cellulose pulp (DP_average_ = 1321) was very close to the DP distribution of the dissolving pulp (DP_average_ = 1469), whereas the DP distribution of market pulp showed a higher proportion of > 2000 DP cellulose chains, and therefore a higher DP_average_ of 2296. The DP distribution of M170 had a much lower proportion of high DP cellulose chains compared to market pulp, dissolving pulp and M120 with a DP_average_ = 574. Both the market pulp and dissolving pulp are bleached pulps that have been subjected to multiple purification and bleaching steps after primary extraction with kraft or sulfite pulping. This indicates that depending on the fractionation conditions, the unbleached cellulose-rich pulps produced by the ionoSolv process can have relatively similar or lower M_w_ and DP_average_ than the bleached wood pulps reported here and in other studies. For example, Palme et al*.* measured the average M_w_ of two dissolving pulps; (i) Scandinavian softwood from a sulfite process was reported to be 283 kDa (DP = 761) and, (ii) *Eucalyptus* pulp produced by a pre-hydrolysis kraft process was reported to be 182 KDa (DP = 581) (Palme et al. 2016). This can be highlighted as a key feature of the ionoSolv process. While this feature might be a disadvantage for conventional cellulose pulp applications (e.g., textiles and paper), it can be an advantage for CNCs-based applications where the use of bleached cellulose substrate (low DP, high cellulose content) or the use of the low DP MCC has proven essential for effective CNC extraction.

The alkaline H_2_O_2_ oxidation caused significant depolymerization of the cellulose chains. The M_w_ of BM120 and BM170 dropped to 38 kDa mol^−1^ and 59 kDa mol^−1^ with a DP_average_ of 234 and 364, respectively. In fact, the low DP cellulose chains or crystallites (DP < 50 and 50 < DP < 100) accounted for approximately 45% of the total amount of cellulose particles. Cellulose depolymerization routes in this method could be due to the cleavage of $\beta$-1,4-glycoside bonds by hydroxyl and/or other radical species formed as side reactions during the alkaline H_2_O_2_ oxidation. Direct depolymerization of cellulose or via the carbonyl groups on the cellulose might also be playing a role (Zeronian and Inglesby 1995; Pouyet et al. 2014). The NaOH alkaline medium may have also facilitated the swelling of the cellulose fibres, which improved their accessibility and depolymerization. The low DP values for cellulose residues are similar to the so-called LODP found in higher plant cellulose after acid hydrolysis (Sirviö 2019). The LODP value is typically used to describe the rapid reduction in DP of cellulose during acid hydrolysis due to the dissolution of the crystallite defects in the cellulose (traditionally considered as amorphous regions) until the DP reaches a constant LODP value as the attack on crystalline regions is much slower. The LODP values are typically in the range of 140 to 200 for bleached wood pulp and 300 for ramie fibre (Habibi et al. 2010). Oxidation processes also have a similar significant reduction in cellulose DP where wood cellulose DP reduced from 1270 to 300–500 after TEMPO-mediated oxidation (Shinoda et al. 2012). The near-LODP sized cellulose residues will be ideal as cellulose substrates for further processing to CNCs or CNFs. Ji et al. have recently investigated applying acid hydrolysis using citric acid to bleached bagasse pulp where the remaining low DP cellulose residue after the hydrolysis was mechanically fibrillated to produce CNFs (Ji et al. 2019).

#### Crystallinity via X-ray diffraction (XRD)

**Fig. 6:**
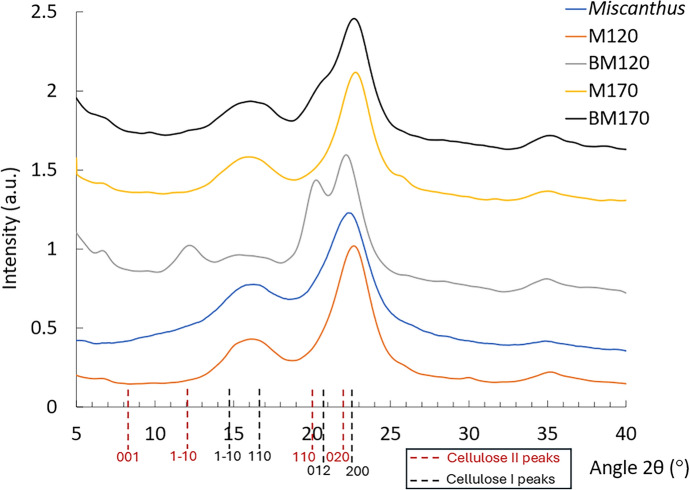
XRD patterns for untreated *Miscanthus*, ionoSolv cellulose pulps and cellulose pulps from the alkaline H_2_O_2_ oxidation. For the staked figure, the intensity of each pattern is depicted by adding a fixed value of intensity to all the data points of the pattern, as follows: M120: + 0 a.u; *Miscanthus*: + 0.2 a.u.; BM120: + 0.5 a.u.; M170: + 1.2 a.u.; BM170: + 1.4 a.u.

The crystalline structure of the cellulose was characterized using XRD and the patterns are presented in Fig. 6. Table 2 presents the CrI values. The crystalline structure of the ionoSolv treated pulps (M120 and M170) remained unchanged from native cellulose I in the untreated biomass with main diffraction angles around 22.5°, 14.5°, and 16.5° (appears as broad signal around 15°) which corresponds to the primary lattice plane (200) and the overlapped peaks from the (1–10) and (110) planes, respectively. Increased intensity of the latter is likely related with an increase in the measure amorphous content (content of crystallite defects). The CrI values of M120 and M170 were 68.7% and 65.18%, respectively which corresponds to crystallinity enhancement of 25 and 20% relative to the untreated *Miscanthus* (51.8%). The increase in crystallinity in the cellulose pulp is related to the removal of the amorphous lignin and branched hemicellulose polymers from the lignocellulose matrix (Tu et al. 2020). The slightly higher crystallinity of M120 compared to M170 can be attributed to the higher delignification of M120, pseudo lignin redeposition on M170 (as suggested by the CA results), and that high severity fractionation conditions attacked some of the crystalline region in cellulose in addition to the crystallite defects region, which is also evidenced by the higher glucan degradation for M170 (Table 1).

**Table 2:** Crystallinity index of ionoSolv treated cellulose pulps and alkaline hydrogen peroxide cellulosic pulps

Sample	CrI (%)
Untreated* Miscanthus*	51.86
M120	68.75
M170	65.18
BM120	59.03
BM170	57.12

The XRD diffraction pattern of the alkaline H_2_O_2_ cellulose oxidation residue BM120 has the characteristic of a mixture of cellulose I and cellulose II as observed by the appearance of cellulose II diffraction angles at 22°, 20°, 12° and 7.5º corresponding to (020), (110), (1–10) and (001) lattice planes, respectively (French 2014). The higher intensity of the 22º peak with respect to the 20º peak, which corresponds with cellulose II (110), indicates overlapping of the cellulose I (200) and cellulose II (020) peaks in the 22º peak (Sèbe et al. 2012). This result aligns with the observations of M120 cellulose dissolution and mercerization during the experiment, causing the cellulose polymorphs to rearrange from cellulose I to cellulose II (Sèbe et al. 2012). However, the XRD pattern of BM170 remained unchanged showing the characteristic cellulose I allomorph diffraction angles. MB170 cellulose pulp also showed an additional peak at 21° corresponding to the (012) plane in a cellulose I allomorph, that could have some contribution from the cellulose II (110) peak. The fact that BM120 and BM170 showed different cellulose polymorphs highlights the different cellulose substrate reactivity levels (M120 and M170) during the alkaline H_2_O_2_ oxidative treatment, which could be related with the higher lignin content of M170. The CrI of cellulose pulps BM120 and BM170 decreased by 14.1 and 12.4%, respectively compared to their precursors (Table 2). The slight decrease in crystallinity indicates that the alkaline H_2_O_2_ oxidative treatment did not only solubilize the crystallite defects regions of the cellulose but it also depolymerizes some of the crystalline high DP cellulose surface, leading to a less crystalline cellulosic pulp. In a conventional TEMPO-mediated oxidation method the crystalline structure of cellulose I is typically maintained during the oxidation, and the carboxylation takes place only in one side of the crystallites surface as the other side of the hydroxy groups is embedded within the crystalline particle (Habibi 2014; Isogai et al. 2018).

#### Solid state ^13^C NMR CP/MAS

Solid state Cross Polarization/Magic Angle Spinning (CP/MAS) ^13^C NMR spectra of raw *Miscanthus*, M120 and BM120 (see SI Figure SI2) show that the lignin in the in the pretreated pulp (M120) has been stripped out as indicated by the loss of the signals in the aromatic regions, the signal for the methoxy group at 55 ppm and the alkyl chains at ca. 22 ppm, which are present in the raw *Miscanthus* and lost in the pretreated pulp. For the bleached sample, the asymmetry for the peak corresponding to C1 at 105.5 ppm is absent on the upfield side of the peak. The solid state ^13^C NMR also allows for the study of the crystallinity and chemical functionalization of the cellulosic samples. The model developed by Newman, in which crystallites are considered to have square cross sections, allows to use the ratio of the areas of C4 and C4’ as an estimation of the weight-averaged lateral dimensions of crystallites, or the fraction of cellulose in the interior of crystallites, with C4 (89 ppm) corresponding with anhydro glucose units in the interior of crystallites and C4’ (84 ppm) corresponding to units in structures with less order or chains in the surface (Newman 1999; Koshani et al. 2018). Therefore, changes in the C4 and C4’ signals have been reported as a proof of the formation of crystalline particles. We observed an increase in the signal intensity of C4 relative to C4’ in the ionoSolv pulp (0.600, see SI table SI3) compared to the raw biomass (0.264), which suggests an increase in crystallinity with post the treatment with IL and the removal of the amorphous lignin and hemicellulose. Further increase in the C4 to C4’ signal ratio was also observed in the bleached pulp BM120 (0.613). The CP/MAS spectra of the bleached BM120 also confirms the formation of type II cellulose by the presence of additional peaks at 107.3 ppm and 63 ppm, suggesting that the sample contains a mix of type Ia /Ib and type II cellulose, which supports the XRD results. Furthermore, there is a reduction in the intensity of the C6 peak, in the region between 60 and 70 ppm, which could be ascribed to the oxidation of the -CH_2_OH groups to form -COOH groups (Koshani et al. 2018).

### CNC characterization

#### Chemical structure and crystallinity

**Fig. 7:**
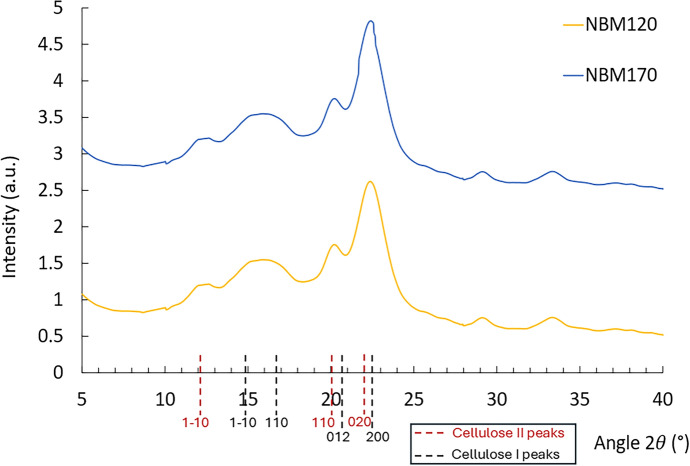
XRD diffraction pattern for freeze-dried CNCs samples NBM170 and NBM120 produced by the alkaline H_2_O_2_ oxidation. For the staked figure, the intensity of each pattern is depicted by adding a fixed value of intensity to all the data points of the pattern, as follows: NBM120: + 0 a.u; NBM170: + 2.0 a.u

The nanocellulose suspensions (as well as their freeze-dried form) obtained by the alkaline H_2_O_2_ oxidation of M120 and M170 were labelled as **NBM120** and **NBM170**, respectively. The yields of the dry nanocellulose matter, with respect the ionoSolv samples M120 and M170 employed as feedstocks for the oxidation, ranged between 12.5 and 28.4 wt% for different experiments with the NBM120 sample and of 2.2 wt% for the NBM170 sample. Solid bleached pulp precipitates were also recovered, with yields ranging between 74.9 and 45.5 wt% for the different experiments with the MB120 sample and 88.7 for the BM170 sample. The mass loss after adding up the CNC and bleached pulp yields should correspond to the solubilization of smaller oligomer chains. The significant difference in yields between both samples is a strong indication that the effect of the pretreatment on the resulting pulps plays a major role in the further stage to produce the CNCs. To improve conversion, a different methodology involving milder H_2_O_2_ oxidation coupled with sonication was developed, as described in the following sections. Figure 7 shows the XRD patterns for NBM120 and NBM170 obtained by H_2_O_2_ oxidation. The diffraction patterns show key characteristics of both cellulose I and cellulose II polymorphs. A sharp peak at $2\theta$ = 22.5° corresponding (200) lattice plane and a broader peak at 15° and 16.5° for the (1–10) and (110) planes found in cellulose I as well as two diffraction peaks at 12° and 20° corresponding to the (1–10) and (110) planes in cellulose II polymorphs. This indicates that some of the CNCs produced in suspension during the alkaline H_2_O_2_ were transformed from native cellulose I to cellulose II, via a mercerization process (Peter 2021). It supports that the transformation of the cellulose allomorph was indeed promoted by the fibre swelling in the alkaline NaOH solution.

The CrI of the NBM170 and NBM120 were determined as 55.2 and 51.5%, respectively. It is interesting to note that both samples showed a lower crystallinity compared to the solid cellulose residue BM170 and BM120 (Table 3). Lower crystallinity of CNCs relative to the cellulose substrate has also been reported when using other oxidation methods such as catalyst-assisted H_2_O_2_ oxidation (Koshani et al. 2018). It is well known that the crystallite defects regions of cellulose are more accessible for dissolution, leaving the crystalline cellulose I behind. The exposure of cellulose I in NaOH solution caused destruction and rearrangement of the intramolecular and intermolecular hydrogen bonds network during the mercerization process, leading to cellulose II (Chen and van de Ven 2016; Koshani et al. 2018). A study of Gong et al*.* investigating the morphology and crystallinity of CNCs extracted from different cellulose sources concluded that the transformation of the cellulose allomorph from cellulose I to cellulose II leads to a reduction in crystallinity (Gong et al. 2017). Lower crystallinity of CNCs relative to the starting cellulose was also reported using other CNC extraction methods. Zhang et al. reported a crystallinity reduction of 20% of cellulose II CNCs extracted from a cellulose I substrate using mercerization in NaOH/thiourea (Zhang et al. 2019). Wang et al. reported a similar low CrI of the CNCs relative to the starting substrate cellulose when extracted by acid hydrolysis using a mixture of H_2_SO_4_/HCl (Neng et al. 2008). The relatively low crystallinity of the obtained CNCs can be improved by carefully optimizing the alkaline H_2_O_2_ oxidation conditions (temperature, time, NaOH and H_2_O_2_ concentration), which will be conducted in a future study. Also, we have developed an alternative, described in the next sections of this document, based on milder H_2_O_2_ oxidation followed by sonication, that helped improving CrI of CNCs. It should be noted that while high crystallinity of CNCs is important in several intended applications (e.g., to improve barrier properties of composite), crystallinity is not critical for other CNCs composite applications where the nanocellulose is mixed with other highly crystalline structures (e.g., graphene oxide) (Lou et al. 2015).

**Table 3:** Composition of the bleached ionoSolv after treatment with a milder alkaline H_2_O_2_ oxidation employing 5 wt% NaOH and 4 wt% H_2_O_2_ at 80 °C and 10 wt% solids loading. Yields of BM170* and BM120* are relative to the amount of M170* and M120, respectively, used in the bleaching experiments

Sample	Pulp Yield	Glucan	Hemicellulose	Lignin	Ash
M120	56.9	84.4	5.1	9.2	1.3
M170*	45.2	87.8	2.7	8.7	0.9
BM120*	86.4	87.9	2.8	8.7	0.6
BM170*	89.0	91.9	4.2	3.9	0.0

The lack of any surface functionality promotes the aggregation of CNCs via hydrogen bonding and Van der Waals forces (Patel et al. 2019). Therefore, chemical functionalization in-situ during the CNC extraction process or in a post treatment is an important prerequisite to an effective use of the extracted CNCs. Sulfuric acid catalysed hydrolysis produces CNCs functionalized by sulfate ester groups, whereas CNCs produced by oxidation methods using TEMPO-mediated, APS or H_2_O_2_ are typically functionalized with carboxyl groups or aldehyde groups (Habibi et al. 2010). The obtained suspensions were highly stable, as no precipitate was observed for over 6 months. This indicates that the nanoparticles generated were small and negatively charged, preventing their agglomeration to larger particles over a long period of time. To assess the colloidal stability of the suspensions, the surface charge of the CNC suspensions was examined using zeta potential (ZP) measurements. Both NBM120 and NBM170 samples showed similar negative ZP of −32 and −31 mV respectively, which is within the common values of CNC obtained by acid hydrolysis and TEMPO oxidation methods (−20 to −40 mV) and slightly higher than the reported ZP values of $\sim$−20 mV using catalyst-assisted H_2_O_2_ and APS oxidization methods (Abraham et al. 2011; Koshani et al. 2018). The ZP values indicate that there is a relatively strong repulsive electrostatic force among the CNCs in suspension due to the presence of the negatively charged groups on the surface. Surface functionality of CNC is very important as it directly impacts its performance in end-use applications (Habibi 2014). High dispersibility of CNCs is critical for applications requiring composite preparation in solvent/polymer matrices.

**Fig. 8:**
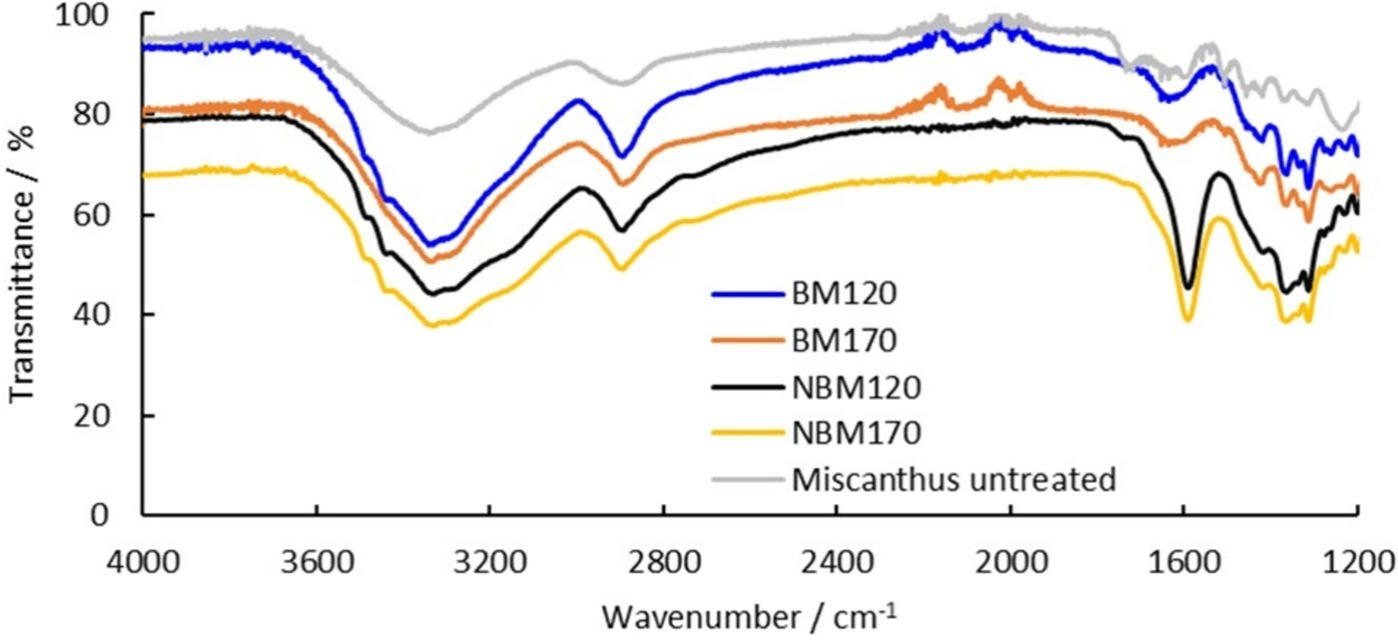
FT-IR spectra of untreated Miscanthus and CNCs samples NBM170 and NBM120 produced by the alkaline H_2_O_2_

To gain further insights on the structure and functionality of the CNCs, FT-IR spectra were recorded and compared to the spectra of the cellulose residues BM120 and BM170 and the untreated *Miscanthus* (Figure 8). Both NBM120 and NBM170 showed new high intensity peak at 1593 cm^−1^ which corresponds to C=O stretching and medium intensity peaks at 1313 to 1364 cm^−1^ which corresponds to characteristic C–O stretching in carboxylate moieties (Zhu et al. 2017; Chen et al. 2019). This confirms that NBM120 and NBM170 contain carboxylated nanocellulose particles. The mechanism of cellulose oxidation by H_2_O_2_ has been reported to be similar to TEMPO-mediated oxidation where carboxyl groups are introduced in the *C*6 position (Zhang et al. 2009; Koshani et al. 2018). It could have gone through a metal-assisted catalysis originating from the metal-rich ash content remaining in the cellulose pulp (ash content of M120 and M170 is 1.3% and 1.8%, respectively Table 1). Analysis of the metal content of the ash residue conducted using XRF shows the presence of various metals and heavy metals (Table SI2 in Supplementary information). It is well-known that the presence of trace metals (e.g., Fe^+2^, Cu^+2^, Mn^+2^) might drive the formation of hydroxyl radicals in H_2_O_2_ oxidation reactions. Equations 1–3 shows a possible radical formation pathway in the presence of a catalytic amount copper ions MCu^+2^ as proposed by Sèbe et al*.* (2012).

1 $${MCu}^{+2}+{H}_{2}{O}_{2} \to {MCu}^{+1}+ {\bullet O}_{2 }^{-}+2{H}^{+}$$

2 $${M}^{+1}Cu+ {H}_{2}{O}_{2} \to {MCu}^{+2}+ \bullet OH+{OH}^{-}$$

3 $${MCu}^{+1}+ {H}_{2}{O}_{2} \to {MCu}^{+2}+ \bullet OH+{OH}^{-}$$

The radicals formed react with the primary hydroxy group at C6 position or secondary hydroxy groups in C2/C3 position during the oxidation, introducing aldehyde and carboxyl groups. Equation 4–6 presents a possible pathway for the oxidation of C6 hydroxy group (Koshani et al. 2018).

4 $$RC{H}_{2}OH+ \bullet OH \to R\bullet CHOH+ {H}_{2}O$$

5 $$R\bullet CHOH+ {H}_{2}{O}_{2} \to RCHO+ \bullet OH+{H}_{2}{O}_{2}$$

6 $$RCHO+ 2 \bullet OH \to RCOOH+ {H}_{2}O$$

The carboxyl band at 1593 cm^−1^ did not appear in the cellulose residue BM120 and BM170, however the medium intensity peak at 1313 to 1364 cm^−1^ corresponding to characteristic C–O stretching of carboxylate was present. This indicates that cellulose residues BM120 and BM170 might have been also potentially oxidized, but presumably to a lower level compared to the CNCs. This may be due to the smaller size of the CNCs, which provides a larger contact surface area to undergo oxidation than the insoluble cellulose residue (Ji et al. 2019). In addition, the appearance of the absorption band in the FT-IR spectrum of both the CNC samples, NBM120 and NBM 170, at 1593 cm^−1^ indicates the presence of a carboxylate group on the CNC surface (Chinga-Carrasco et al. 2023). The absorption band in carboxylic acids typically appears at 1730 cm^−1^ and is not to be found in our samples. The presence of the deprotonated carboxylate is expected since the oxidation was conducted at highly alkaline conditions (pH ~ 11).

#### Morphology and dimension

**Fig. 9:**
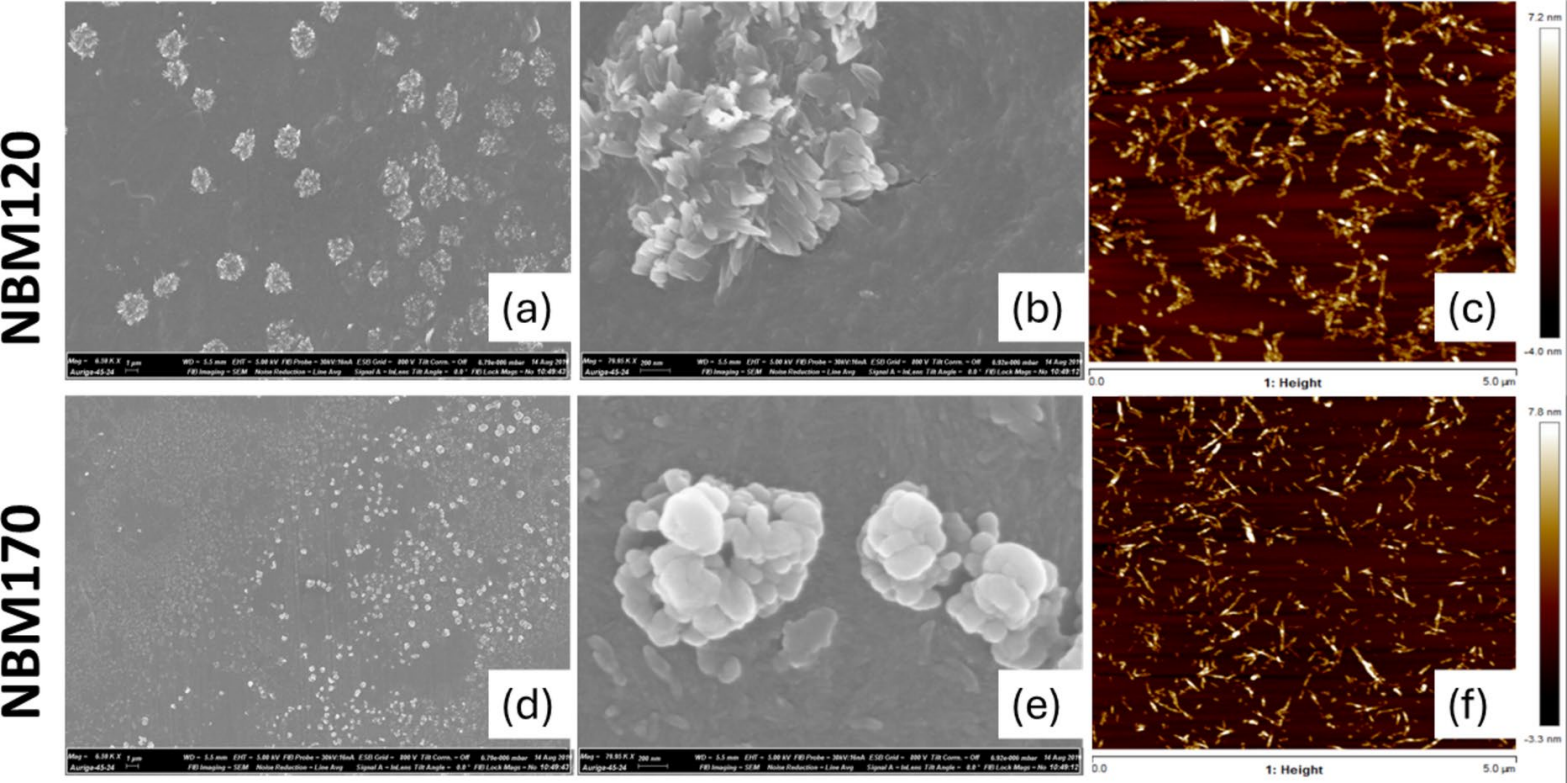
Images of NBM120 (Top) and NBM170 (bottom). **a** low magnification SEM of NBM120, **b** high magnification SEM of NBM120, **c** AFM height image for NMB120, **d** low magnification SEM of NBM170, **e** high magnification SEM of NMB170, **f** AFM height image for NMB170 (bottom). Full size SEM and AFM images are provided in the ESI (figs SI8 to SI13)

The morphology of the obtained CNCs in the cellulose suspension after freeze drying was observed using SEM (Fig. 9a,d). Large groups of bundles of nanoparticles were clearly visualized. These bundles are likely to have been formed in-situ during sample drying, as is to be expected on these types of materials, especially since no additional dispersing agents were used (Stinson-Bagby et al. 2018). A similar alignment of nanorods bundles was observed by Koshani et al*.* for copper assisted H_2_O_2_ oxidation for CNC isolation from softwood pulp (Koshani et al. 2018). It is interesting to observe the topographical morphology differences at higher magnification between the two samples. The nanocellulose bundles of NBM120 look rod-like whereas NBM170 nanocellulose look quasi-spherical (Fig. 9b,e). Well-dispersed CNCs could be seen clearly from the AFM images, which are a better representation of the individual particles in solution (Fig. 9c,f). Both NBM120 and NBM170 showed a needle-like CNCs, with dimensions below 30 nm width and up to 300 nm in length, and the CNCs were fairly individualized, which supports that the crystallite aggregates observed by SEM were mostly induced by the in-situ drying during sample preparation.

#### Thermal stability and UV transmittance

**Fig. 10:**
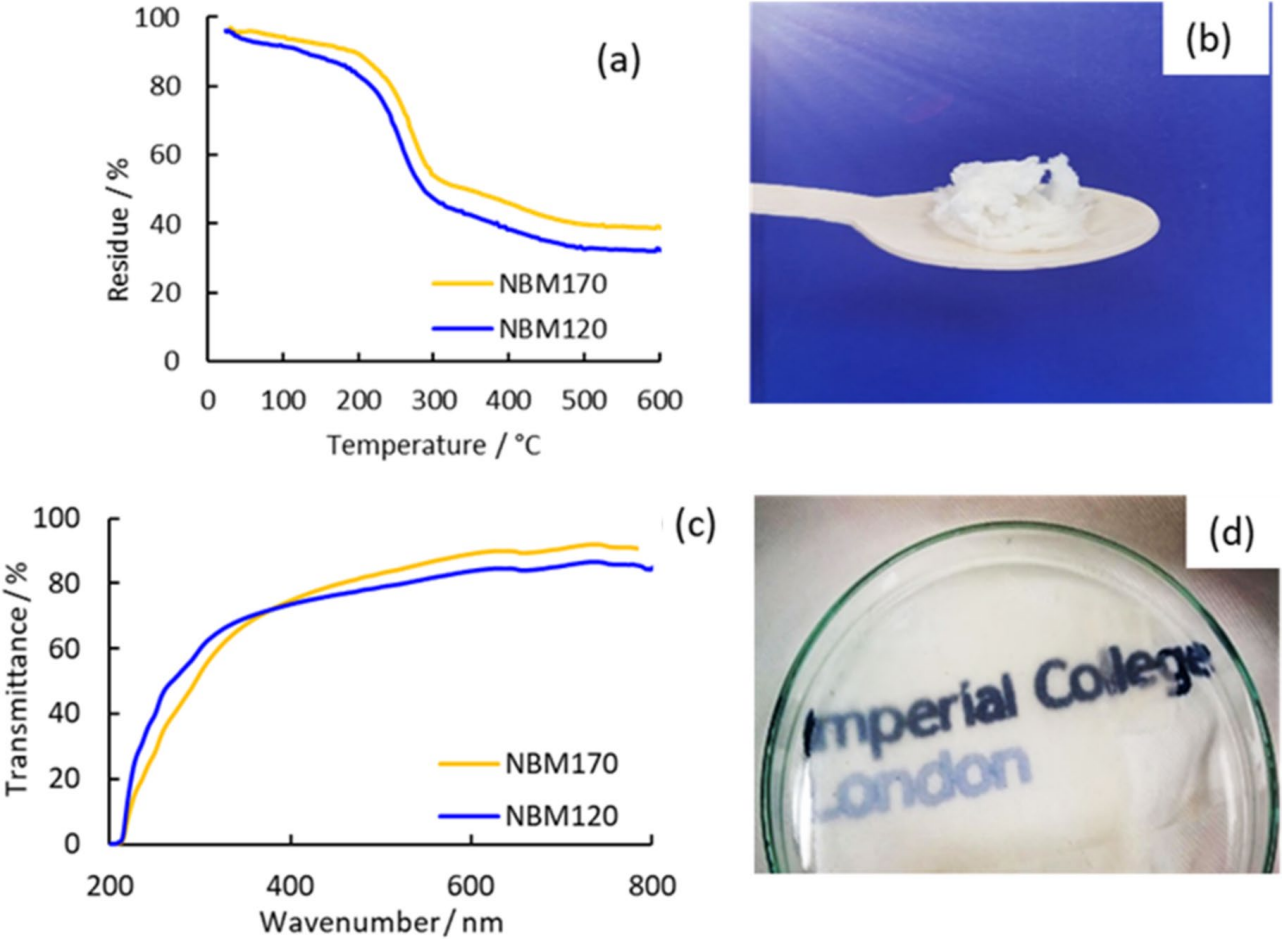
**a** TGA curves for freeze-dried CNCs, **b** freeze-dried CNCs suspension, **c** UV–vis spectrum for CNCs suspensions, **d** nanofilm formation on petri-dish

Thermal stability of CNCs is an important property for several potential applications e.g. composites for electronic devices and fire retardants (Bajwa et al. 2019; Norrrahim et al. 2021). Figure 10a shows the TGA curves for the obtained CNCs. The weight loss under 150 °C can be attributed to water evaporation. NBM120 showed slightly higher thermal stability, with T_0_ of 230 °C, compared to 210 °C for NBM170. These temperatures are within the T_0_ range typically obtained for TEMPO-oxidized CNC/CNFs. The lower thermal stability of CNCs compared to the substrate cellulose pulps (typically T_0_ > 250 °C) is related to the significant reduction of DP and, more importantly, to the introduction of the carboxyl groups on their surface, which are thermally unstable and naturally hydrophilic (Lichtenstein and Lavoine 2017; Chen et al. 2019). Furthermore, the somehow large degradation between 100 and 200 °C might be related to the presence of some sulfate groups, arising from the use of a hydrogensulfate based IL as pretreatment method, and known to decrease thermal stability of CNCs (Roman and Winter 2004; Wang et al. 2007). The char content of NBM120 and NBM170 after thermal decomposition were 32% and 38%, respectively. The high char content highlights the significant influence of surface functionalization on the thermal stability of the obtained CNCs and it is within the range of char content obtained from other oxidized CNCs. Lichtenstein and Lavoine studied the thermal degradation mechanism of TEMPO-oxidized CNFs (Lichtenstein and Lavoine 2017). The study showed that the surface chemistry of the oxidized CNFs had a significant impact on the lower thermal stability compared to non-oxidized CNFs. The ash content of the TEMPO-oxidized CNFs was 10 to 20% higher compared to the non-oxidized CNFs, which has been attributed to the extensive change in surface chemistry (Lichtenstein and Lavoine 2017).

**Fig. 11:**
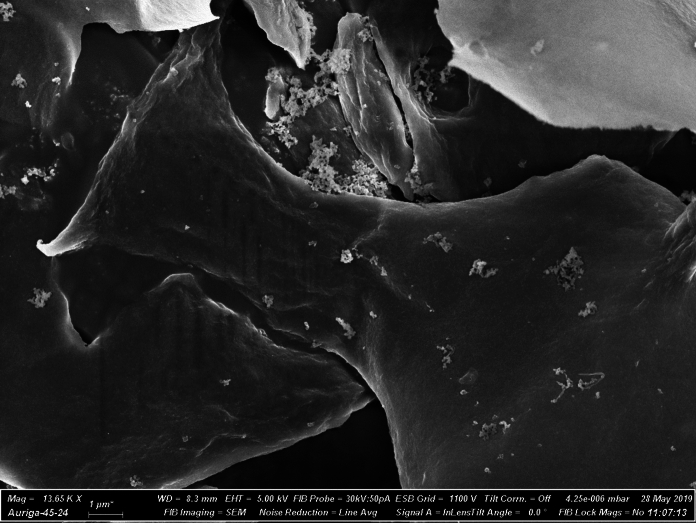
SEM of freeze dried CNCs

While many CNC applications are based on their use in reinforced composites, there has been growing interest on the use of CNCs as self-standing films or aerogels and foams. Freeze drying the CNC suspensions produced ultra-light white foam (Fig. 10b) which were characterized by SEM to show an amorphous architecture with mushroom-like morphology (Fig. 11). On the other hand, casting the CNC suspensions on petri dish at ambient temperature formed a nanofilm. Because of the dense packing of the CNCs, the nanofilm was highly transparent (Fig. 10d). Figure 10c shows the UV–vis spectrum of the CNC aqueous suspensions NBM120 and NBM170, both with an optical transparency of 0.1 wt. The optical transparency of NBM120 and NBM170 at 600 nm were 83% and 89%, respectively, similar to those of previous reports (Tan et al. 2016; Wang et al. 2020). The transmittance of the CNC solution in the UV–vis spectrum is a result of both light absorption and scattering, which can be affected by the chemical structure and the size of the CNCs particles. As the particles in NBM120 suspension were larger than the particles in NBM170, it is expected that the amount of light scattered also will be higher, resulting in the observed decrease of optical transmittance.

## Production of CNCs by ultrasonication

To improve the yields of CNC production, a modified methodology that involves the use of ultrasonication was implemented. For this, harsh oxidation is not needed during the bleaching stage and a milder bleaching protocol was implemented (as described in the materials and methods section).

Color removal was not as intense as with the previous experiments and, interestingly, was more effective for M170 than for M120. The composition of the pulps also reflects that bleaching with this milder process was not as effective in removing lignin as that employed previously. However, the purity and composition of the resulting BM170* (we will be referring to the newly produced samples with an *) was very close to that of the previous BM120 and BM170, while allowing to significantly reduce the usage of H_2_O_2_ (4 wt% relative to the pulp weight vs 150 wt%). Furthermore, the resulting DP_average_ was higher than with the previous method for both bleached pulps: 632 for BM120* and 640 for BM170*.

The bleached pulps were then ultrasonicated at different times (15 to 60 min) to analyze the effect of the ultrasonication time in the CNC yield. The ultrasonicated samples were labelled as NBM120UM, and NBM170UM according to the ultrasonication conditions, wherein M denotes the ultrasonication time in minutes.

It was observed that ultrasonication of BM170* for 60 min yielded a stable suspension that showed no traces of precipitation after 48 h of settling and centrifugation for 30 min at 3000 rpm, and no traces of solid residue (figure SI4). On the other hand, ultrasonication of BM170* for 15 min and of BM120* for both 15 and 60 min yielded 2-layer systems in which a lighter CNC suspension is floating over a jelly-like material (figure SI4).

The CrI value of UNBM170U60 as measured by X-ray diffraction was 63%, higher than that of NBM170 (55.2%), BM170 (58.7%), and close to that of the starting ionoSolv pulp M170 (65.18%). Also, the diffraction patterns of the NBM170U60 sample showed the preservation of the cellulose I structure, with a narrower peak at 15° corresponds to (1–10) planes, and a strong peak at 2 = 22.5° belongs to (200) lattice plane, and no trace of the cellulose II characteristic peaks at 12° and 20° that correspond to (1–10) and (110) planes (figure SI5). This suggests that the milder oxidation procedure helped preserving the original crystallinity structure, which was not altered significantly during the ultrasonication process.

The milder oxidation conditions are also reflected by the FT-IR spectrum. The carboxyl band at 1593 cm^−1^ found for the CNCs prepared using strong oxidation conditions is not found in the spectra of the CNCs prepared by ultrasonication after milder bleaching procedure (figure SI6).

ZP of NBM170U60 was recorded with a value of −38 mV (table SI4), even lower to that of the CNCs obtained by oxidation (−31 mV for NBM170). Again, suggesting the presence of negatively charged surface in the obtained nanoparticles in suspension, leading to repulsive interactions between them and preventing their aggregation. It should be noted that the ZP values of the 2 layers obtained after 15 min of ultrasonication were significantly different from each other (−41 mV for the gel-like lower phase and −28 mV for the low concentration CNC dispersion in the upper phase).

Production of CNCs by ultrasonication also seemed to increase the thermal stability of the material, with NBM170U60 showing a T_0_ of 270 °C and NBM120U60 a T_0_ of 230 °C (figure SI7). The increased thermal stabilities could be related to the higher DP of the pulps bleached under the milder oxidation conditions and to a lower degree of surface substitution due to the reduced exposure to the oxidizing agents.

## Conclusions

This study demonstrates a simple one-step alkaline H_2_O_2_ oxidation procedure to produce carboxylated nanocellulose using unbleached cellulose substrates produced by a protic IL-based fractionation process. We showed that the severity of the IL fractionation has a significant impact on the cellulose composition and purity and, consequently, in on its reactivity with the oxidating reagents. The more delignified pulp M120 showed higher mercerization effect compared to M170, as the cellulose allomorph changed from cellulose I to cellulose II after the alkaline oxidation process. The results also showed that cellulose substrates produced from the fractionation have relatively low Mw and DP compared to commercially bleached pulp. The subsequent alkaline oxidation procedure also resulted in significant reduction of the Mw and DP of cellulose producing a near-LODP cellulose residue that can be further processed to CNFs or CNCs using few processing steps. Analysis of the cellulose-rich suspensions showed the generation of negatively charged, electrostatically stable, needle-like CNCs. The crystallinity of the CNCs was lower compared to their precursors, which indicates the high level of degradation of the crystalline regions of the cellulose. Further control over the oxidation reagents and IL fractionation conditions could potentially improve the crystallinity of the CNCs generated. The produced CNCs showed the attachment of carboxyl functional groups, which explains the stability of the suspension. The carboxylation of the CNC surfaces caused a slight decrease in their thermal stability, which is a known effect when using oxidation methods to produce CNCs.

To maximize the production of CNCs, a milder oxidation process employed just to reduce lignin content followed by ultrasonication was also tested. Ultrasonication for 60 min allowed total conversion of the bleached pulps into CNCs. The reduced demand for oxidizing agent also allowed for better preservation of the cellulose crystal structure during the process and to an increased thermal stability.

## Supplementary Information

Below is the link to the electronic supplementary material.

Supplementary file1 (DOCX 7008 KB)

## Data Availability

Data is provided within the manuscript or supplementary information files.
