# Supplementary material for: Efficient extraction of carboxylated nanocellulose from ionoSolv pulps with alkaline H2O2 assisted oxidation
Source: Cellulose (Lond). 2024 Dec 11;32(2):853–75. doi: 10.1007/s10570-024-06319-4 (PMC11774951; doi:10.1007/s10570-024-06319-4)
Supplement: Supplementary file 1 — Supplementary file1 (DOCX 7008 KB) [file 10570_2024_6319_MOESM1_ESM.docx]

Efficient Extraction of Carboxylated Nanocellulose using ionoSolv process with Alkaline H_2_O_2_ assisted Oxidation

Aida Abouelela Rafat^a^, Pedro Verdía Barbará^a^, Asim Ullah^a^, Eero Kontturi^b^, Rob V. Law^c^, Jason P. Hallett^a,*^

^a^ Department of Chemical Engineering, Imperial College London, South Kensington, Exhibition Road, SW7 2AZ, London, United Kingdom

^b^ Department of Bioproducts and Biosystems, Aalto University, P.O Box 16300, 00076 Aalto Espoo, Finland

^c^ Department of Chemistry, Imperial College London, Molecular Sciences Research Hub, White City Campus, 82 Wood Ln, W12 0BZ, London, United Kingdom

* Corresponding author, email address: [j.hallett@imperial.ac.uk](mailto:Jason.hallett@imperial.ac.uk)

**ORCID of the authors**

Aida Abouelela Rafat: -

Pedro Verdía Barbará: 0000-0001-6471-6305

Asim Ullah: -

Eero Kontturi: 0000-0003-1690-5288

Rob V. Law: 0000-0001-6710-8110

Jason P. Hallet: 0000-0003-3431-2371

**Electronic Supplementary Information**

**Table of Contents**

[**Biomass fractionation** 2](#_Toc171412887)

[**Chemical compositional analysis** 2](#_Toc171412888)

[**Independent analysis of the composition of pulps using an IR methodology** 4](#_Toc171412889)

[**Impact of the fractionation severity on the pulp brightness** 4](#_Toc171412890)

[**Metal content in the *Miscanthus* biomass feedstock used in this study** 4](#_Toc171412891)

[**Solid state ^13^C NMR of ionoSolv and bleached pulps** 6](#_Toc171412892)

[**Bleaching of ionoSolv pulps with mild H_2_O_2_ treatment** 7](#_Toc171412893)

[**Production of CNC by ultrasonication** 7](#_Toc171412894)

[**SEM and AFM images** 10](#_Toc171412895)

[**References** 14](#_Toc171412896)

# **Biomass fractionation**

Fractionation experiments were carried out in triplicates, according the standard procedure of our group (Gschwend et al., 2016). An IL solution to biomass ratio of 1:5 g/g (corresponds to 2±0.1 g of air-dry biomass) and 10±0.05 g IL-water mix were added into a wide-mouthed 40 mL Ace pressure tube with screw cap and shaken with a vortex shaker to ensure all biomass was fully wetted. The pressure tube was sealed, vortexed and then introduced to the oven at a predetermined temperature for specific period of time. The cellulose-rich pulp was separated from the ionic liquid slurry, washed 3 times using 40 mL of high purity ethanol in 50 mL Falcon tube. The tube was shaken for 1 min and left to settle at room temperature for at least 1 hour. The tube was shaken again for 1 min and centrifuged at 3,000 rpm for 50 min. Followed, the cellulose pulp was further washed using 24 h Soxhlet extraction with ethanol. Following Soxhlet extraction, the thimbles containing pulp were emptied in pre-weighted falcon tube and washed with 30 mL of water to remove traces of ethanol and ensure that pulp is kept wet in water. After decanting the wash water, the weight of the falcon tube and wet pulp is recorded for pulp yield calculation. Approximately 1 g of the wet pulp was taken for moister content measurement. Pulp yield was calculated as follows:

$$Pulp yield= \frac{m_{pulp x (1-moisture content)}}{m_{oven-dried biomass}} x 100$$

# **Chemical compositional analysis**

Compositional analysis was carried out according to the published procedure ‘Determination of Structural Carbohydrates and Lignin in Biomass’ by the NREL (Sluiter et al., 2012). Ceramic crucibles were placed into a muffle oven (Nabertherm + controller P 330) and ashed 2 to 3 times at muffle oven at 575°C until constant weight is reached. 300 mg (calculated on ODW basis) of air-dry biomass or recovered biomass was weighed out into a pressure tube and the weight recorded (Sartoriaum CPA 1003 S balance, ±0.001 g). 3 mL of 72% sulfuric acid was added, the samples stirred using a Teflon stir rod and the pressure tubes placed into a preheated water bath at 30°C. The samples were stirred every 10 min for one hour. Samples were then diluted with 84 mL distilled water and the lids closed. The samples were autoclaved (Sanyo Labo Autoclave ML5 3020 U) for 1 hour at 121°C and left to cool to close to ambient temperature. The samples were then filtered through filtering ceramic crucibles of a recorded weight. The filtrate was filled in two 15-mL Falcon tubes and the remaining black solid washed with distilled water. The crucibles were placed into a convection oven (VWR Venti-Line 115) at 105°C for 24±2 hours. They were then taken out and placed in a desiccator for 15 min before they were weighed, and the weight of the AIL plus the crucible were recorded. The content of acid insoluble lignin (AIL) was determined according to the following equation:

$\%AIL=\frac{\left( {Weight}_{crucible plus AIR}-{Weight}_{crucible} \right)-{(Weight}_{crucible plus ash}-{Weight}_{crucible})}{{ODW}_{sample}}x100$

The content of one of the Falcon tubes was used for the determination of acid soluble lignin content (ASL) by UV analysis at 240 nm (equation 2) (Perkin Elmer Lambda 650 UV/Vis spectrometer)

$\%ASL=\frac{{UV}_{abs}x {Volume}_{filtrate}xDilution}{\varepsilon x {ODW}_{sample}} x 100$

where UV_abs_ is the average UV-vis absorbance for the sample at 240 nm; Vfiltrate is 86.73 mL; the dilution factor is the ratio of total diluted sample volume to undiluted sample volume, as needed to bring the absorbance within the range 0.7-1.0; and ε is the biomass absorptivity (12 L g-1cm^-1^ for pine).

The other Falcon tube contents were neutralized by careful addition of calcium carbonate until the pH reached 5. After settling, the liquid was filtered through a 0.2 μm PTFE syringe filter and submitted to HPLC analysis for the determination of total sugar content. HPLC analysis of glucose, xylose, mannose, arabinose and galactose was performed on a Shimadzu HPLC with an AMINEX HPX-87P Column (Biorad, 300 x 7.8 mm, prepacked HPLC carbohydrate analysis column) with refractive index detection. The mobile phase was de-ionized water, the column temperature 85°C and the flow rate was 0.6 ml min^-1^. Calibration standards with concentrations of 0.1, 1, 2 and 4 mg mL^-1^ of glucose, xylose, mannose, arabinose and galactose and 8 mg mL^-1^ of glucose were used. Sugar recovery standards were made as 10 mL aqueous solutions close to the expected sugar concentration of the samples and transferred to pressure tubes. 278 µL 72% sulfuric acid was added, the pressure tube closed and autoclaved and the sugar content determined as described above. The sugar recovery coefficient (SRC) was determined according to equation 7 and the sugar content of the analyzed sample using

$SRC=\frac{C_{HPLC}x V}{inital weight}$

The sugar concentration values obtained by HPLC were corrected to obtain the percent hydrolyzed sugar recovery values, accounting for any dilution made prior to HPLC analysis, using the following equation:

$\%Sugar=\frac{C_{HPLC} x V x {corr}_{hydro}}{SRC x ODW}$ x100

where C_HPLC_ is the sugar concentration determined by HPLC (mg/mL), V is the dilution factor (86.73 mL for samples and 10 mL for SRC standards). Corr_hydro_ is the correction for the mass increase during hydrolysis of polymeric sugars (0.90 for C6 sugars glucose, galactose and mannose and 0.88 for C5 sugars xylose and arabinose) and ODW is the oven-dried weight of the sample in mg. SRC is the sugar recovery standard value for each sugar. The sugars content in the pulp were normalized based on the pulp yield obtained in the pretreatment as:

${Glucan}_{pulp}= \frac{C_{x}}{pulp yield x mass closure}$

# **Independent analysis of the composition of pulps using an IR methodology**

To assess the deviation of the compositional analysis results for the pulps with ultra-low content of cellulose, 3 selected samples, one from our bleached ionosolv pulps and two commercial pulp samples were sent for analysis to Celygnis Analytical. Results are shown in the table below.

**Table SI1**. Composition of pulps using IR methodology

| **Sample Name** | **Total Sugars** | **Glucan** | **Xylan** | **Mannan** | **Arabinan** | **Galactan** | **Klason Lignin** | **Acid Soluble Lignin** | **Extractives** | **Ash** |
| --- | --- | --- | --- | --- | --- | --- | --- | --- | --- | --- |
| DISSOLVING PULP | 93.36 | 88.35 | 4.76 | 0.23 | 0.02 | - | 0.92 | 0.83 | - | 0.00 |
| MARKET PULP | 93.49 | 80.61 | 6.32 | 5.72 | 0.60 | 0.24 | 3.20 | 0.88 | - | 0.52 |
| MB170 | 91.58 | 90.87 | 0.42 | 0.20 | 0.04 | 0.05 | 0.99 | 0.66 | - | 1.49 |

# **Impact of the fractionation severity on the pulp brightness**


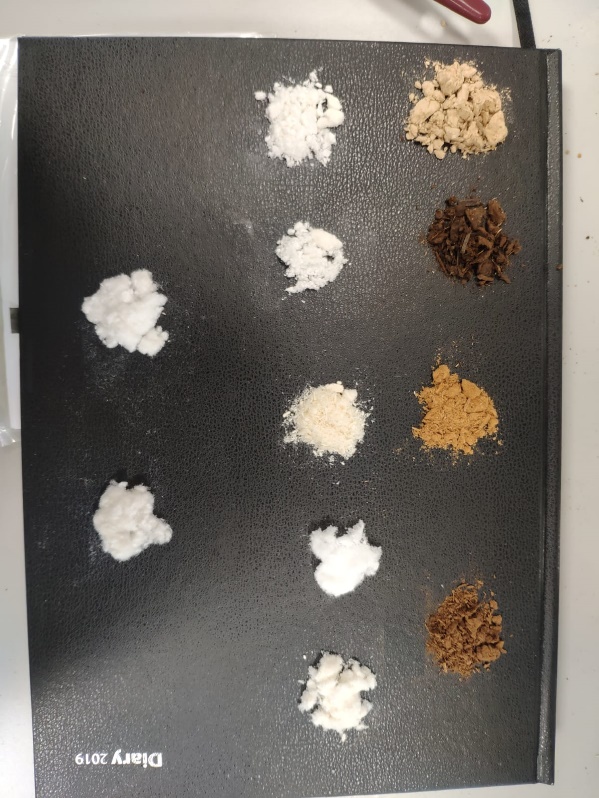

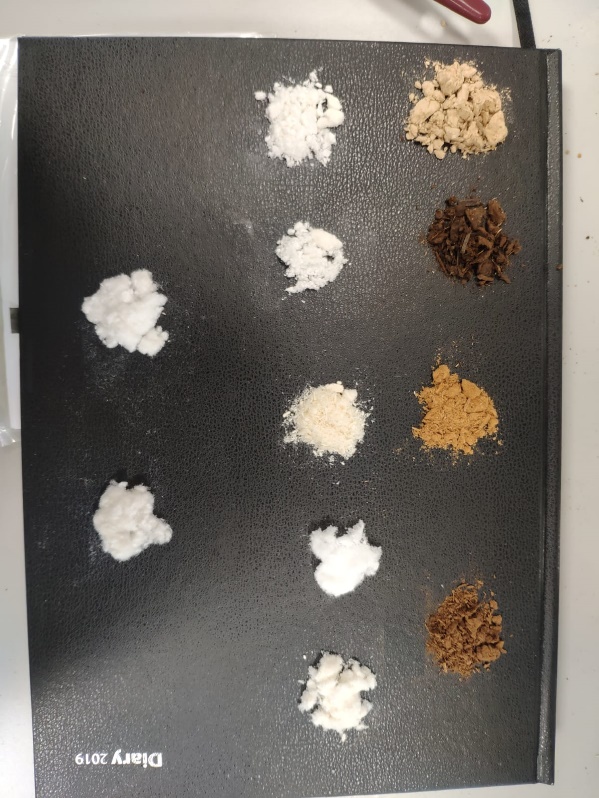

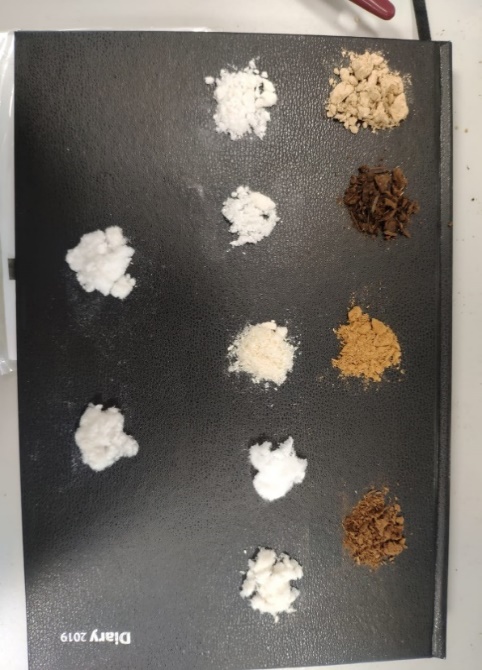

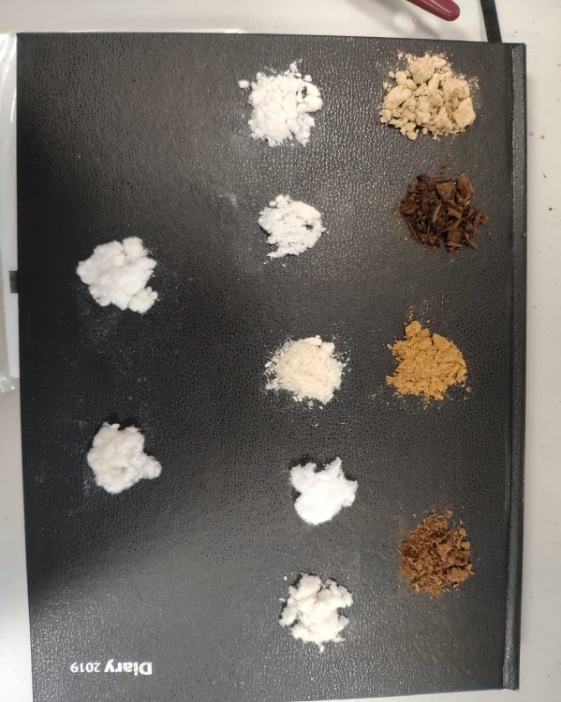

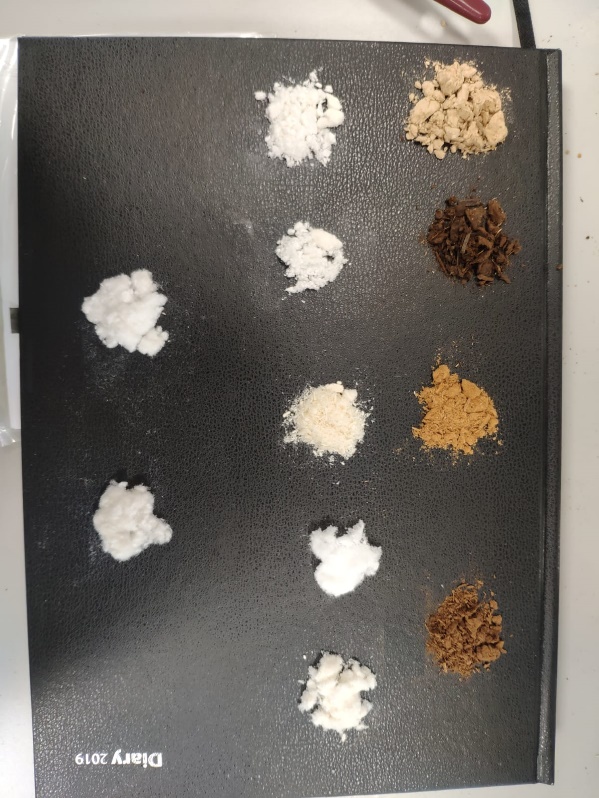

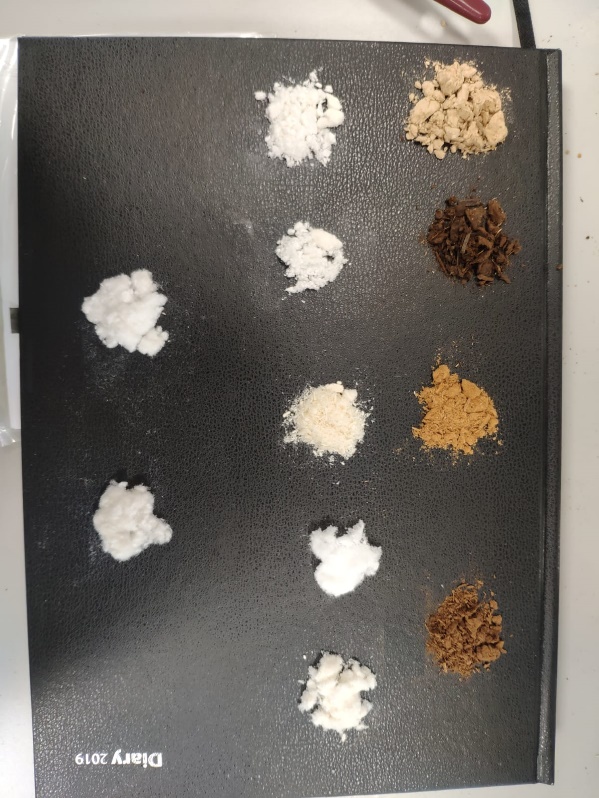


M120

M170

BM120

BM170

Dissolving pulp

Market pulp

**Fig SI1** Impact of the fractionation conditions on the cellulose pulp brightness

# **Metal content in the *Miscanthus* biomass feedstock used in this study**

**Table SI2**. Composition of the ash content of Miscanthus biomass, as determined by XRF methodology. The Miscanthus ash was produced by heating samples of *Miscanthus X Giganteus* raw biomass up to 575 °C in a muffle oven.

| Miscanthus ash | | |
| --- | --- | --- |
| Compound | Concentration | Unit |
| Mg | 1,122 | % |
| Si | 9,59 | % |
| P | 3,219 | % |
| Cl | 857,1 | ppm |
| K | 4,407 | % |
| Ca | 2,101 | % |
| Ti | 34,8 | ppm |
| Cr | 3,6 | ppm |
| Mn | 589,2 | ppm |
| Fe | 145.4 | ppm |
| Ni | 1,6 | ppm |
| Cu | 10.3 | ppm |
| Zn | 60,3 | ppm |
| As | 0 | ppm |
| Br | 0,3 | ppm |
| Rb | 0,4 | ppm |
| Sr | 4,4 | ppm |
| Zr | 0 | ppm |
| Ce | 34,7 | ppm |
| Nd | 0 | ppm |
| Yb | 0,7 | ppm |
| Pb | 0,6 | ppm |
| SO3 | 0,1 | % |
| O | 79,293 | % |
| Ag | 781,246 | cps |
| S | 5,137,005 | cps |

# **Solid state ^13^C NMR of ionoSolv and bleached pulps**

**
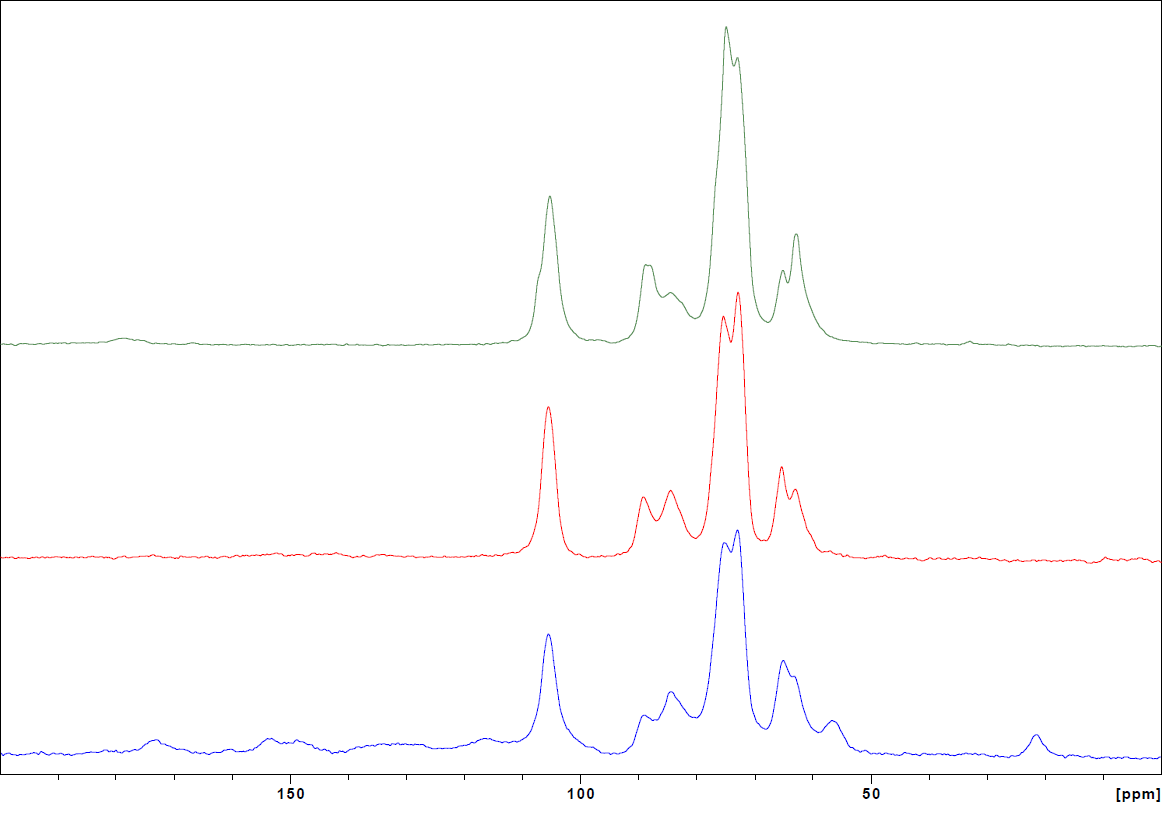
**

**Fig SI2** Solid state ^13^C NMR spectra of the bleached *Miscanthus* pulp BM120 (green, top) *Miscanthus* pulp M120 (red, middle) and the corresponding raw *Miscanthus* (blue, bottom)

**Table SI3**. Peak fitting for the C4 and C4’ signals of raw *Miscanthus,* ionosolv pulp M120 and bleached BM120 using Lorezian/Gauss functions after being normalised and calculated as a percentage of the total.

| Raw *Miscanthus* |  |
| --- | --- |
| C4 | **0.209** |
| C4' | **0.791** |
| M120 |  |
| C4 | **0.375** |
| C4' | **0.625** |
| BM120 |  |
| C4 | **0.380** |
| C4' | **0.620** |

# **Bleaching of ionoSolv pulps with mild H_2_O_2_ treatment**


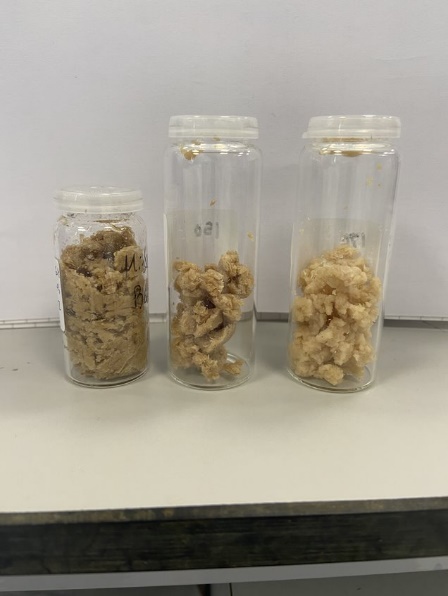


Fig SI3 Resulting wet pulps after bleaching with 4 wt% of H_2_O_2_ relative to biomass ODW the ionoSolv pulps pretreated at 120 ºC for 6 hours (BM120*, left), 150 ºC for 65 minutes (BM150*, centre) and 170 ºC for 45 minutes (BM170*, right)

# **Production of CNC by ultrasonication**


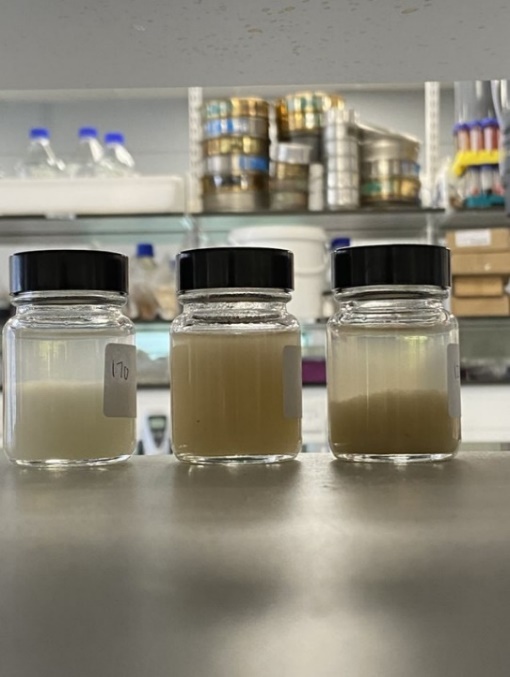

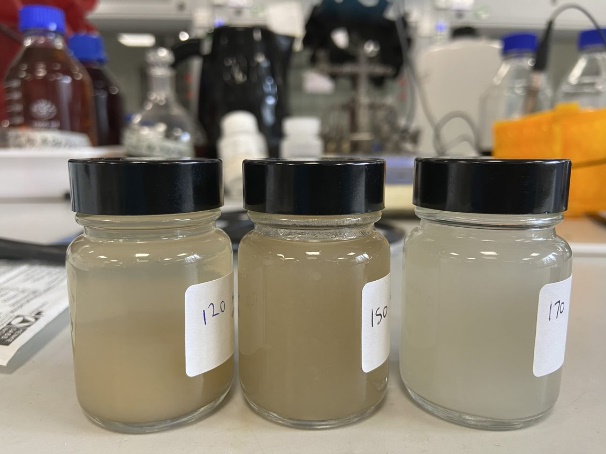


Fig SI4 Top: bleached pulps ultrasonicated for 15 minutes. From left to right: NBM120U15, NBM150U15, and NBM170U15. Bottom: Bleached pulp ultrasonicated for 60 minutes. From left to right: NBM120U60, NMB150U60, and NMB170U60


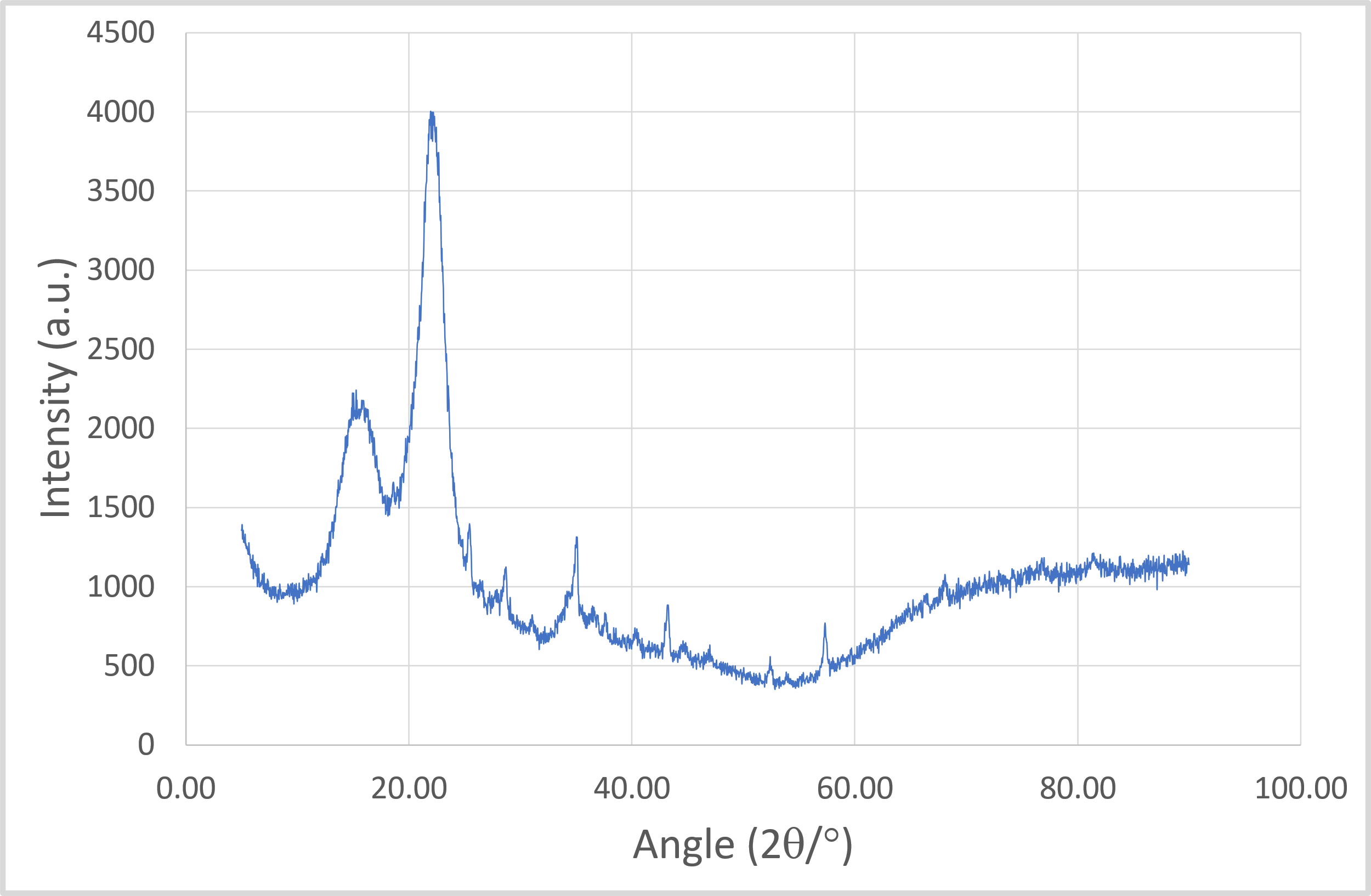


**Fig SI5** XRD diffraction pattern for the freeze-dried CNCs sample NBM170U60


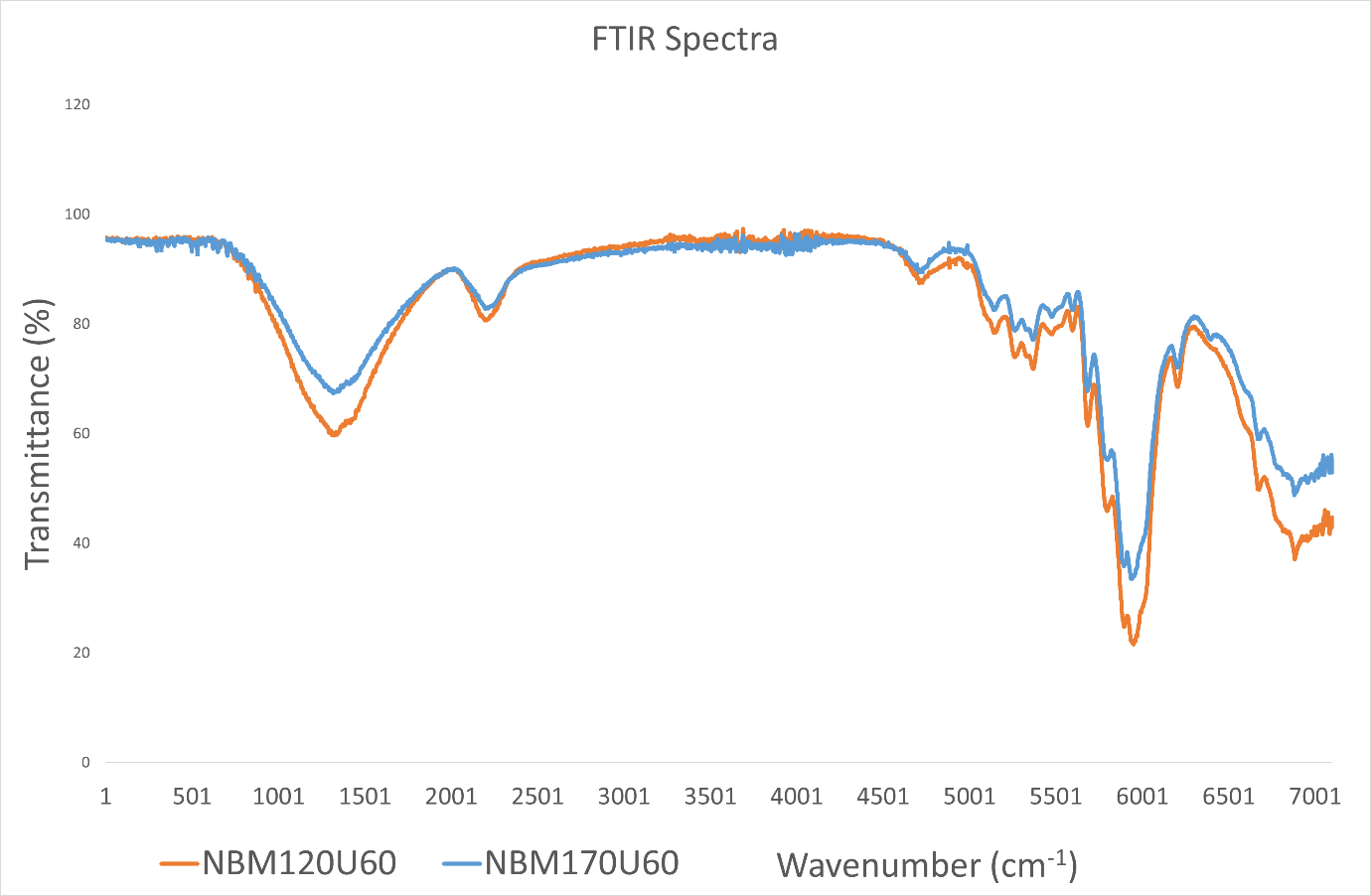


**Fig SI6** FTIR Spectra of CNC samples obtained by ultrasonicating for 60 minutes bleached ionoSolv pulps obtained after pretreatments at 120 min for 6 h (NBM120U60, orange), and 170 ºC for 45 minutes (NBM170U60, blue)

**Table SI4**. Zeta potential values for the CNC suspension samples obtained after ultrasonication.

| Sample | Mean zeta potential (mV) |
| --- | --- |
| NBM170U15 - lower phase | -41.38489598 |
| NBM170U15 - upper phase | -28.05523769 |
| NBM170U60 | -38.96894472 |


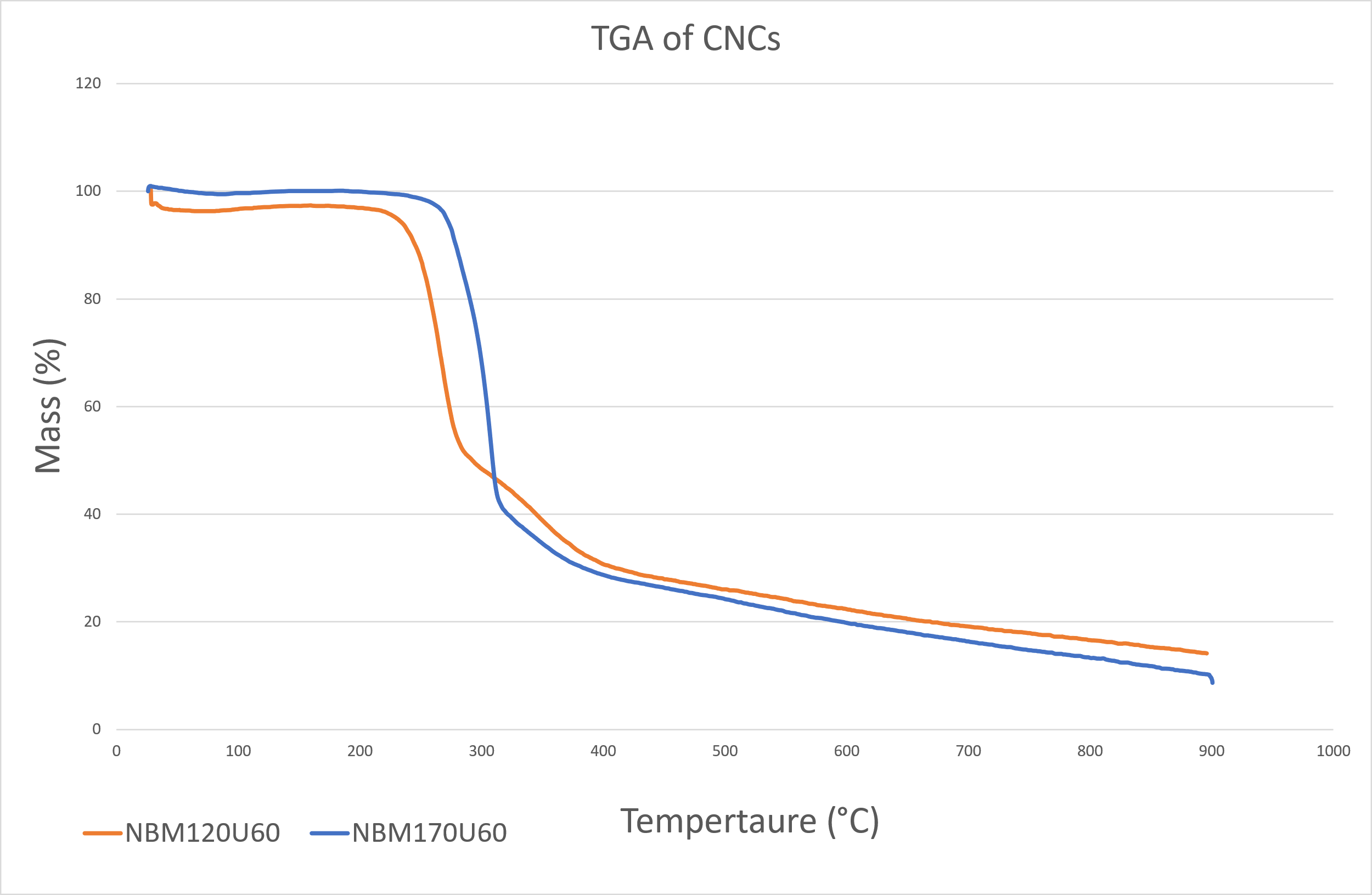


**Fig SI7** TGA of of CNC samples obtained by ultrasonication for 60 minutes of ionoSolv pulps obtained after pretreatments at 120 min for 6 h (NBM120U60, orange), and 170 ºC for 45 minutes (NBM170U60 blue)

# **SEM and AFM images**


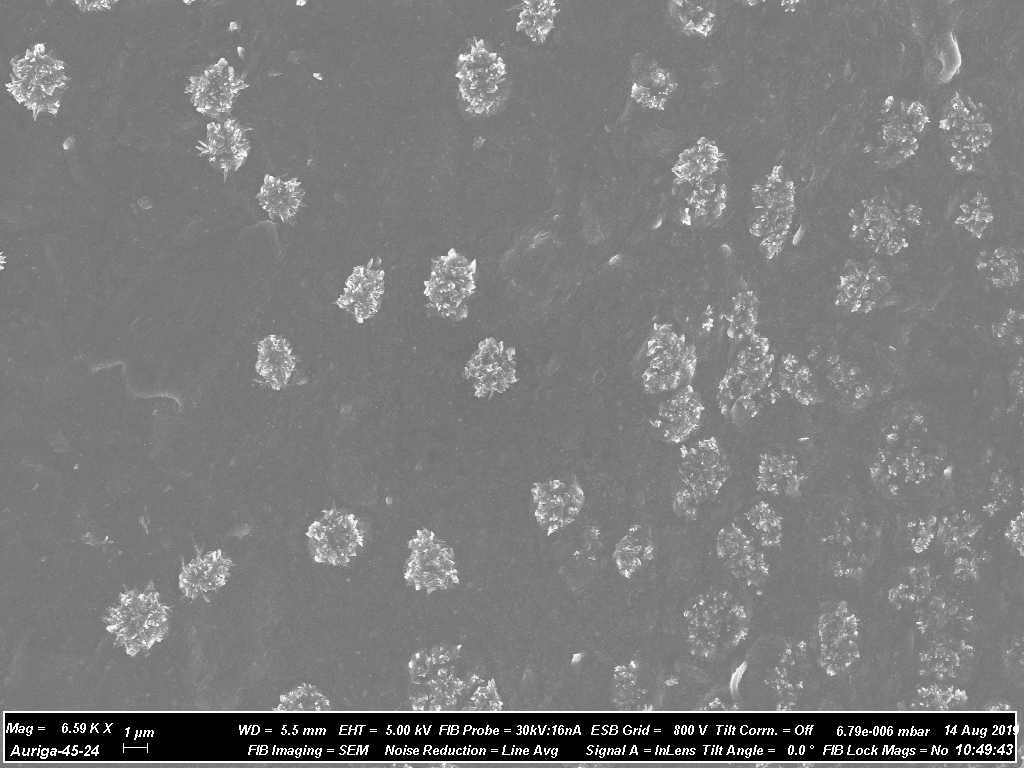


**Fig SI8** SEM image of NBM120 at low magnification.


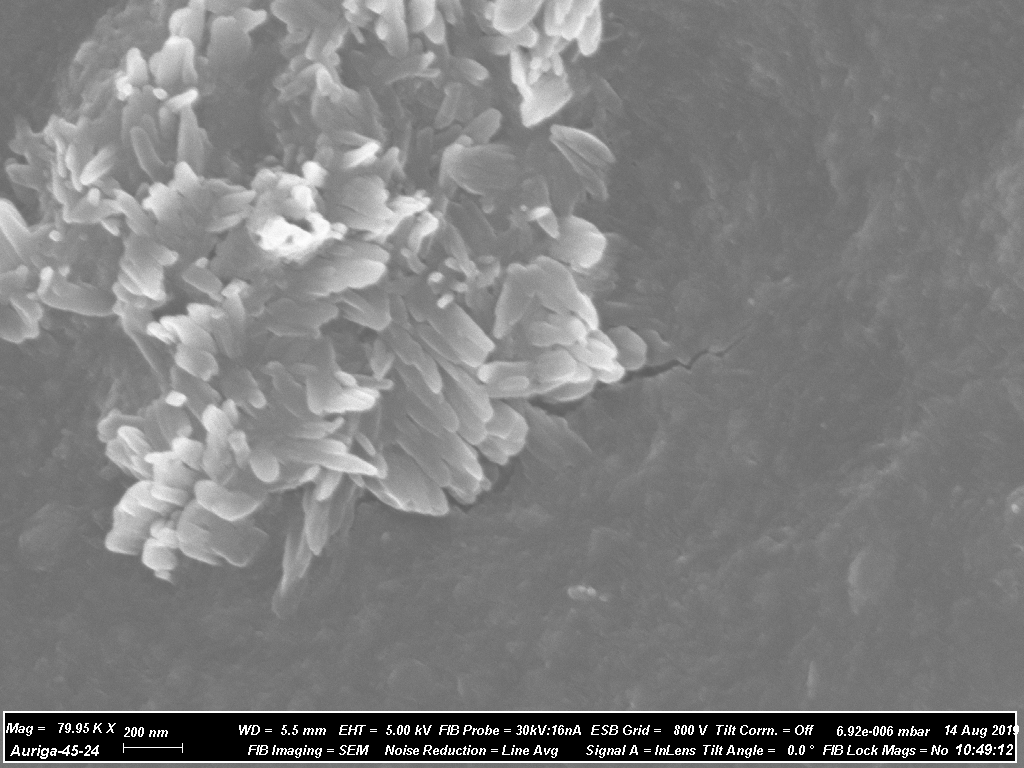


**Fig SI9** SEM image of NBM120 at high magnification


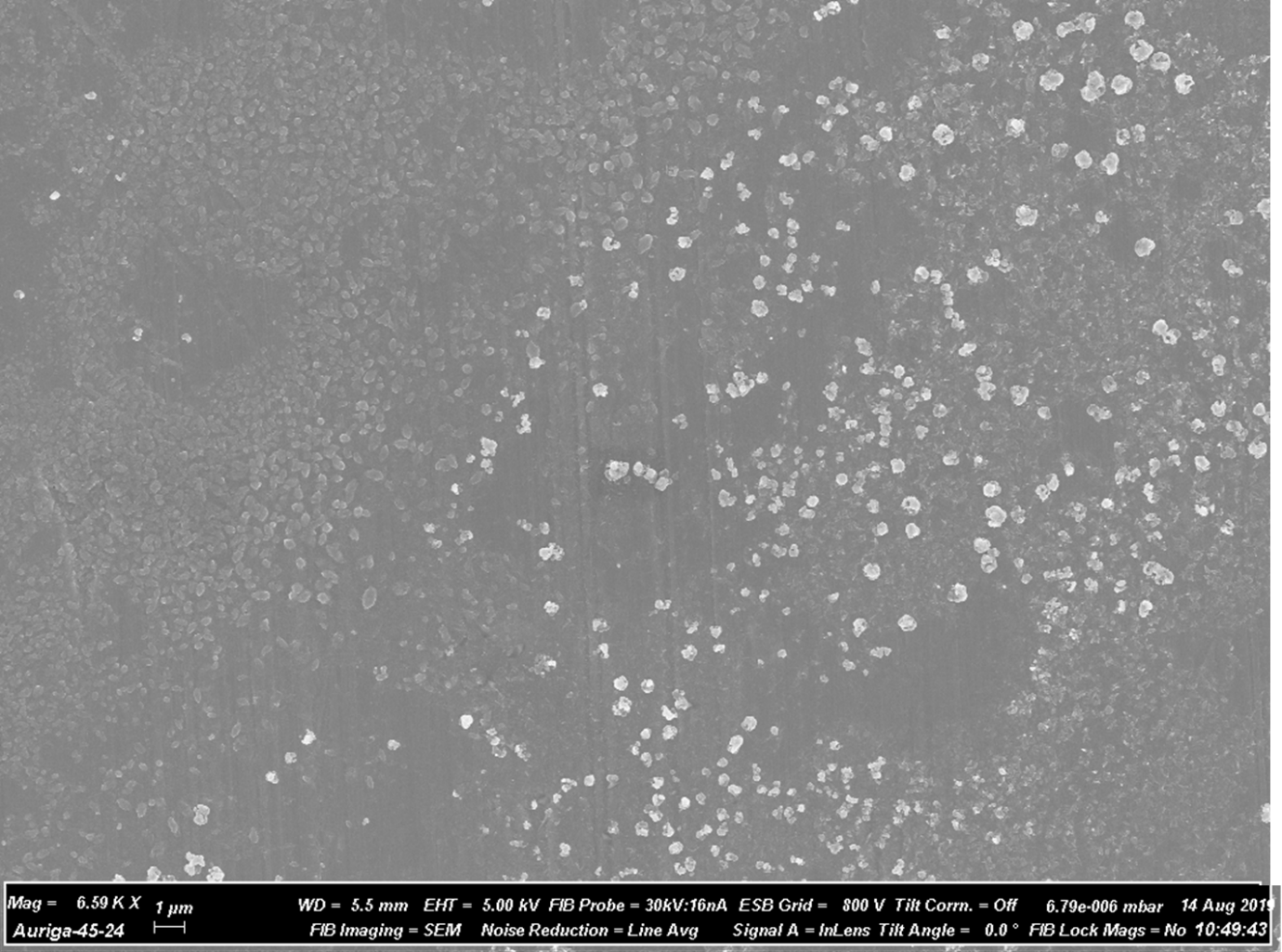


**Fig SI10** SEM image of NBM170 at low magnification


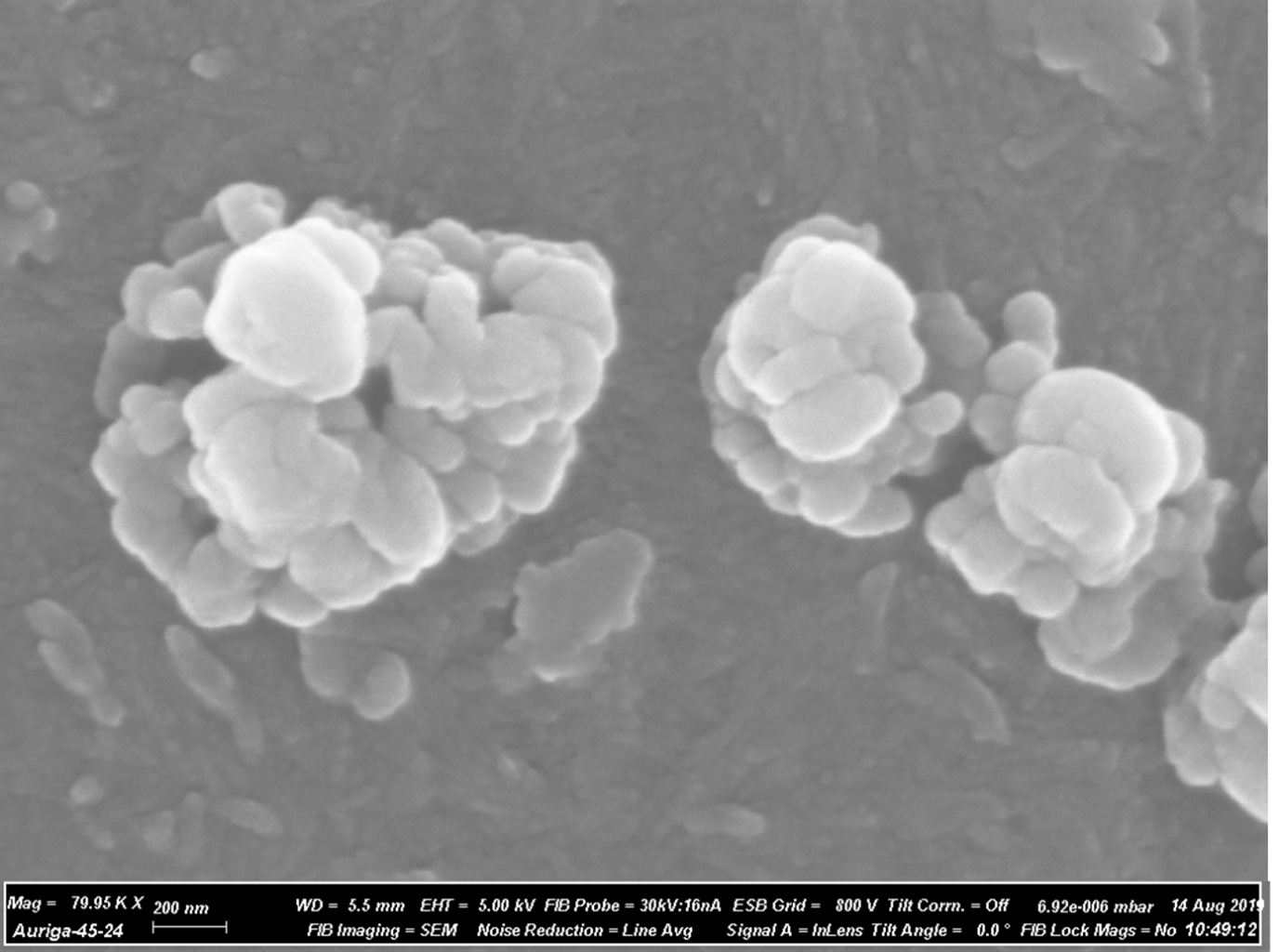


**Fig SI11** SEM image of NBM170 at high magnification


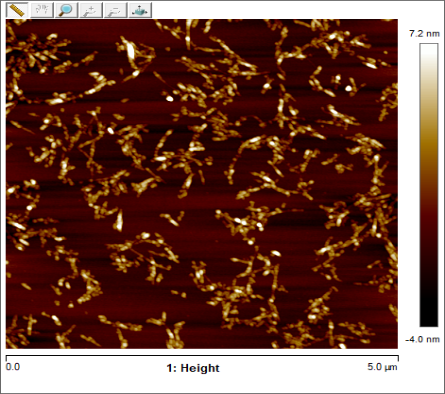


**Fig SI12** AFM height image for NMB120


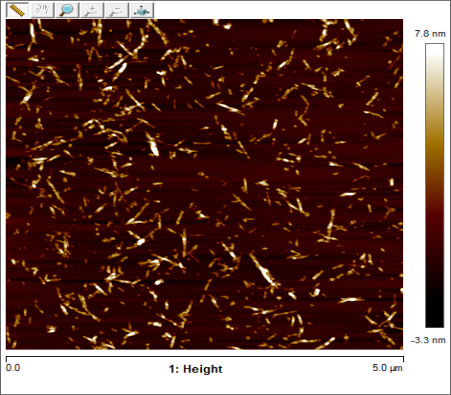


**FIG SI13** AFM height image for NMB170

# **References**

Gschwend, F. J. V, Brandt, A., Chambon, C. L., Tu, W.-C., Weigand, L., & Hallett, J. P. (2016). Pretreatment of Lignocellulosic Biomass with Low-cost Ionic Liquids. *JoVE*, *114*, e54246. https://doi.org/doi:10.3791/54246

Sluiter, a., Hames, B., Ruiz, R., Scarlata, C., Sluiter, J., Templeton, D., & Crocker, D. (2012). Determination of structural carbohydrates and lignin in Biomass. In *Laboratory Analytical Procedure (LAP)* (Issue April 2008). https://doi.org/NREL/TP-510-42618
